# A computational approach to design a multiepitope vaccine against H5N1 virus

**DOI:** 10.1186/s12985-024-02337-7

**Published:** 2024-03-20

**Authors:** Fatemeh Dashti, Arash Raisi, Ghazaleh Pourali, Zahra Sadat Razavi, Fatemeh Ravaei, Javid Sadri Nahand, Fatemeh Kourkinejad-Gharaei, Seyed Mohammad Ali Mirazimi, Javad Zamani, Hossein Tarrahimofrad, Seyed Mohammad Reza Hashemian, Hamed Mirzaei

**Affiliations:** 1 School of Medicine, Kashan University of Medical Sciences, Kashan, Islamic Republic of Iran; 2 Student Research Committee, Kashan University of Medical Sciences, Kashan, Islamic Republic of Iran; 3 Metabolic Syndrome Research Center, Mashhad University of Medical Sciences, Mashhad, Islamic Republic of Iran; 4 Infectious and Tropical Diseases Research Center, Tabriz University of Medical Sciences, Tabriz, Islamic Republic of Iran; 5 Department of Infectious Diseases, Emam Reza Hospital, Sirjan School of Medical Sciences, Sirjan, Islamic Republic of Iran; 6 Department of Animal Biotechnology, National Institute of Genetic Engineering and Biotechnology (NIGEB), Tehran, Islamic Republic of Iran; 7 Chronic Respiratory Diseases Research Center (CRDRC), National Research Institute of Tuberculosis and Lung Diseases (NRITLD), Shahid Beheshti University of Medical Sciences, Tehran, Islamic Republic of Iran; 8 Research Center for Biochemistry and Nutrition in Metabolic Diseases, Institute for Basic Sciences, Kashan University of Medical Sciences, Kashan, Islamic Republic of Iran

**Keywords:** Docking, Immunoinformatics, Influenza, Molecular dynamics, Vaccine

## Abstract

Since 1997, highly pathogenic avian influenza viruses, such as H5N1, have been recognized as a possible pandemic hazard to men and the poultry business. The rapid rate of mutation of H5N1 viruses makes the whole process of designing vaccines extremely challenging. Here, we used an in silico approach to design a multi-epitope vaccine against H5N1 influenza A virus using hemagglutinin (HA) and neuraminidase (NA) antigens. B-cell epitopes, Cytotoxic T lymphocyte (CTL) and Helper T lymphocyte (HTL) were predicted via IEDB, NetMHC-4 and NetMHCII-2.3 respectively. Two adjuvants consisting of Human β-defensin-3 (HβD-3) along with pan HLA DR-binding epitope (PADRE) have been chosen to induce more immune response. Linkers including KK, AAY, HEYGAEALERAG, GPGPGPG and double EAAAK were utilized to link epitopes and adjuvants. This construct encodes a protein having 350 amino acids and 38.46 kDa molecular weight. Antigenicity of ~ 1, the allergenicity of non-allergen, toxicity of negative and solubility of appropriate were confirmed through Vaxigen, AllerTOP, ToxDL and DeepSoluE, respectively. The 3D structure of H5N1 was refined and validated with a Z-Score of − 0.87 and an overall Ramachandran of 99.7%. Docking analysis showed H5N1 could interact with TLR7 (docking score of − 374.08 and by 4 hydrogen bonds) and TLR8 (docking score of − 414.39 and by 3 hydrogen bonds). Molecular dynamics simulations results showed RMSD and RMSF of 0.25 nm and 0.2 for H5N1-TLR7 as well as RMSD and RMSF of 0.45 nm and 0.4 for H5N1-TLR8 complexes, respectively. Molecular Mechanics Poisson-Boltzmann Surface Area (MM/PBSA) confirmed stability and continuity of interaction between H5N1-TLR7 with the total binding energy of − 29.97 kJ/mol and H5N1-TLR8 with the total binding energy of − 23.9 kJ/mol. Investigating immune response simulation predicted evidence of the ability to stimulate T and B cells of the immunity system that shows the merits of this H5N1 vaccine proposed candidate for clinical trials.

**Supplementary Information:**

The online version contains supplementary material available at 10.1186/s12985-024-02337-7.

## Introduction

A member of the Orthomyxoviridae family is influenza virus A [1]. This virus is responsible for causing acute respiratory disease and consistently changes while circulating among various animal hosts, such as wild birds, pigs, poultry, and horses as well as humans. The differentiation of these viruses is primarily according to the surface glycoproteins, specifically Hemagglutinin (HA) along with Neuraminidase (NA) [2]. There exist eighteen distinct variations of HA as well as eleven different NAs, each of which can be distinguished serologically. Notably, antibodies produced against a virus genotype don’t exhibit a reaction through other genotypes [3].

H5 influenza viruses have been typically found in avian populations and are naturally hosted by birds. However, they can infect a range of mammal species as well. There are nine recognized subtypes of H5 viruses, which include H5N1, H5N3, H5N2, H5N5, H5N4, H5N7, H5N6, H5N9 and H5N8. Among these subtypes, Worldwide, the viruses known as Low Pathogenic Avian Influenza (LPAI) are the most often seen in poultry and wild birds. Nevertheless, Highly Pathogenic Avian Influenza (HPAI) viruses are sporadically identified as well. The very first known instance of a highly pathogenic H5N1 virus infection in humans was reported by Yuen et al. 1998 [4]. These H5N1 viruses have significantly spread across regions in Asia, and the world. They have undergone rapid evolution, giving rise to ten distinct clades, labeled as clades 0 through 9. Since 2007, H5N1 viruses have been identified to be a significant hazard to men, wild birds, as well as poultry. Specifically, clade 2.2.2 was prevalent from 2007 to 2011, and from 2011 onwards, clade 2.3.2.1a has been in circulation [5].

The genotypes of H5N1 are determined by eight single-stranded RNA segments that encode a total of 11 proteins, among them surface glycoproteins known as neuraminidase (NA) and hemagglutinin (HA) [6]. Both HA and NA play essential roles that revolve around their contact with sialic acid. A terminal structure seen on glycoproteins or glycolipids that are expressed on the surface of cells is sialic acid [7]. The mechanism known as clathrin-mediated endocytosis, which is facilitated by HA's attachment to sialic acids on cellular receptors, allows the virus to enter cells. It's worth noting that the virus may also employ alternative endocytic pathways, like micropinocytosis, for cell entry [8].

NA is important in the final phases of infection. In particular, it functions to eliminate sialic acids from both cellular receptors and the freshly produced NA and HA present upon developing virions. The glycosylation activities of the host cell include the addition of these sialic acids [9, 10]. Moreover, NA's cleavage of sialic acids inhibits virions from clumping together and from attaching themselves to dying host cells via HA. This, in turn, facilitates the efficient release of virions and their spread to new target cells [9]. Given their crucial roles in the virus's pathogenicity, In the realm of vaccine research and development, HA and NA have become the main targets to neutralize avian influenza viruses (AIV) [10–12].

At present, there isn’t a vaccine strain on the market that can successfully produce protective immunity against the various clades and subclades of highly pathogenic avian influenza viruses (HPAIV) in the H5 lineage [13]. Nonetheless, Previously, research has indicated that vaccination against seasonal influenza A strains like H1N1 and H3N2 may improve cross-type cellular and humoral protection against the highly pathogenic avian influenza virus subtype H5N1 [14]. Both our research and the previously mentioned findings underscore the significant public health threat posed by H5N1 viruses, even though the number of human cases may be relatively small.

Consequently, the development of vaccines holds promise in helping to mitigate the impact of disease outbreaks. Reverse vaccinology is a recent breakthrough that, in addition to the conventional and laborious vaccine creation approaches, integrates immunogenomics and bioinformatics to quickly generate unique multi-epitope-based subunit vaccines. This approach offers the potential to expedite the vaccine development process [15, 16]. Today, there are extensive studies based on the use of bioinformatics and immunoinformatics tools to design new-generation vaccines [17–23].

Therefore, the current research aimed to produce a chimeric vaccine versus avian influenza A virus subtype H5N1 that is non-allergenic and immunogenic. This was achieved by utilizing a vaccinia’s approach, and It is advised that model animals be used in a laboratory environment to carry out the experimental validation of the suggested vaccination candidate.

## Material and method

### Antigen retrieval

The Influenza NCBI Research Database included the HA and NA protein sequences from several influenza H5N1 virus strains as described by Squires et al. 2012 [24].

### Liner B cell epitopes and discontinues B cell epitopes prediction

To identify B cell antigenicity, Researchers used the Immune Epitope Database (IEDB) and BepiPred-2.0, a tool available at http://www.cbs.dtu.dk/services/BepiPred/, to detect linear B cell epitopes within the sequences of HA and NA proteins to predict B cell epitopes with 75% accuracy, 0.49 sensitivity, and 0.75 specificity, as detailed in the studies by Saadi et al. (2017) and Saha and Raghava [25](2006a). These tools incorporated several algorithms such as Emini surface accessibility prediction, and Bepipred linear epitope prediction 2.0 as well as Kolaskar and Tongaonkar antigenicity scale, which can be accessed at http://tools.iedb.org/bcell/. The semi-empirical Kolaskar and Tongaonkar technique takes into account the physicochemical characteristics of amino acid residues and their frequency in experimentally established segmental epitopes to predict antigenic determinants on proteins [26].

We also used the ElliPro server to forecast the discontinuous B cell epitopes' three-dimensional shape, which can be accessed at http://tools.iedb.org/ellipro/. ElliPro is a program that takes the three-dimensional structure of the protein into account to help anticipate conformational or discontinuous B cell epitopes. This is crucial for understanding how these epitopes are identified via the immunity system as well as for designing effective vaccines or therapeutic antibodies.

### MHC-I epitope prediction

The T cell epitopes’ prediction, specifically cytotoxic T cell epitopes, has been carried out utilizing an enhanced neural network methodology offered via the NetMHC-4.0 server, accessible at https://services.healthtech.dtu.dk/service.php?NetMHC-4.0. In this analysis, all epitopes have been regarded to be 9-mers in length, as recommended by the server. These 9-mer epitopes are recognized by HLA Class I molecules, including HLA-A, HLA-C as well as HLA-B.

### MHC-II epitope prediction

The epitope binding to MHC-II molecules has been predicted using the NetMHCII 2.3 server. This service predicts epitope affinity for a variety of HLA-II molecules, such as HLA-DR, HLA-DQ, and HLA-DP, using an artificial neural network. By using this prediction tool, Researchers can evaluate the probability that epitopes will attach to MHC-II molecules, which is critical for understanding and designing immune responses, particularly those involving helper T cells.

### Population coverage determination

The Population Coverage instrument in the IEDB server, accessible at http://tools.iedb.org/population, was used to determine the population's coverage rate for particular epitopes. Determining the population coverage is essential to forecast the right epitopes for various HLA-binding interactions. Epitopes that exhibit strong binding to a variety of HLA alleles can provide broader coverage across different ethnicities, reducing the constraints of MHC limitations on T cell reactions. In such context, the evaluation of MHC-I HLA binding included a range of HLA alleles such as HLA-B5701, HLA-B5801. HLA-B5802, HLA-B2705, HLA-A0203, HLA-A2603, HLA-B8301, HLA-B4002, HLA-A3002, HLA-A2902, HLA-B4402, HLA-C0401, and HLA-C0303 for the NA and HA proteins. Additionally, MHC-II HLA binding assessments were performed for DRB1_0101, DPA10201-DPB10501, DPA10103-DPB10301, DQA10501-DQB10201, DRB1_0403, DPA10103-DPB10401, DRB1_0404, DRB1_0403, DRB1_0402, DQA10102-DQB10501, DRB1_0401, DRB1_1302, DQA10301-DQB10302 and DQA10401-DQB10402 for the NA and HA proteins. This comprehensive analysis helps in identifying epitopes that have the potential to generate immunity reactions across a broad range of human populations.

### Analysis of epitopes conservation

The degree of conservation of particular epitopes was evaluated using the IEDB Conservancy Analysis tool as a monitoring tool about other sequences that were comparable. Epitopes can exhibit varying levels of conservation, ranging from a minimum of 0 percent to a maximum of 100 percent, depending on their specific length and sequence. This analysis is important in understanding how well-conserved an epitope is within a given protein or among related sequences. Highly conserved epitopes are more likely to be effective in stimulating immune responses because they are present in a broader range of strains or related proteins, making them potentially useful for vaccine design or therapeutic development. On the other hand, less conserved epitopes may be strain-specific and may have limited utility in generating immune responses across different variants or strains.

### Physicochemical, antigenicity, allergenicity, toxicity properties of the vaccine

In the design of the vaccine construct, various linkers were strategically employed to connect different components and epitopes. These linkers serve as connectors between different functional elements and help optimize the vaccine’s overall design. AAY linkers, specific HEYGAEALERAG linkerGPGPG linkers, KK linkers and EAAAK linkers are used to bond Bcell, CLT and HLT epitopes. Also,HβD-3 and PADRE are used as adjuvants in the N-terminal of the construct.

The ProtParam instrument, available at https://web.expasy.org/protparam/, has been utilized for predicting the physicochemical characteristics of the final protein construct. It provides information about various protein parameters, such as molecular weight, and isoelectric point (pI), as well as amino acid composition.

The VaxiJen v2.0 server, accessible at http://www.ddg-pharmfac.net/vaxijen/VaxiJen.html, has been utilized for predicting the antigenic properties of the protein construct. It's considered an independent alignment method and is employed for antigen prediction. The server's accuracy typically ranges from 70 to 89%. AllerTOP v. 2.0, found at https://www.ddg-pharmfac.net/AllerTOP/, has been utilized for calculating the total protein sensitivity. This server uses an auto cross-covariance (ACC) as well as a k-nearest neighbor algorithm (kNN, k = 1) for predicting the proteins’ allergenicity. It considers various factors, including hydrophobicity, molecular weight, secondary structure characteristics, as well as the amino acids’ relative abundance. The ToxDL server, available at http://www.csbio.sjtu.edu.cn/bioinf/ToxDL/index.html, has been used to assess the protein's toxicity. This server employs an interpretable deep learning-based methodology for categorizing proteins into 2 categories: toxic and non-toxic. It uses a multi-modal approach like Convolutional Neural Networks (CNNs), and the InterProscan database. These tools and servers collectively provide valuable insights into physicochemical characteristics, antigenicity, allergenicity, along toxicity of protein construct, which are essential considerations in vaccine development and protein engineering.

The use of NetSurfP-2.0 online software, available at https://services.healthtech.dtu.dk/service.php?NetSurfP-2.0, has been implemented to predict the secondary structure of the protein. NetSurfP-2.0 is a sequence-based instrument that offers predictions regarding the secondary structure of amino acids within a protein.

### 3D modeling, refinement and validation of the H5N1 construct

The RoseTTAFold online software, accessible at https://robetta.bakerlab.org/, has been employed for constructing the three-dimensional (3D) structure of the protein. RoseTTAFold is considered a sophisticated “three-track” neural network, which means it regards multiple aspects simultaneously for predicting protein structures that have high accuracy. RoseTTAFold considers patterns in protein sequences, allowing it to understand the amino acid sequences that make up the protein.

To guarantee the precision and excellence of the protein structure simulation, PROCHECK and SAVES online servers, available at http://services.mbi.ucla.edu/PROCHECK and http://services.mbi.ucla.edu/SAVES, respectively, were used for structural validation.

The PROSA server, accessible at https://prosa.services.came.sbg.ac.at/prosa.php, has been utilized to identify the protein energy and Z-Score balance. This helps assess the quality and stability of the 3D structure of the protein.

The Ramachandran plot, which depicts the phi as well as psi angles of amino acids in protein structure, was generated using the online website http://molprobity.biochem.duke.edu/. It provides insight into the quality of the protein's backbone torsion angles.

The optimum spatial resolution for the study's optimal energy was found using the PyMol program. It is likely used for visualizing and analyzing the 3D protein structure. The website http://galaxy.seoklab.org/cgi-bin/submit.cgi?type=REFINE has been employed for protein structure refinement.

### Docking analysis

The 3D structures of TLR7 (PDB code: 7CYN) and TLR8 (PDB code: 3w3g), were obtained from the RCSB server. The TLRs’ 3D structures have been adjusted for energy using the PyMOL v2.3.4 program. The vaccination and TLR PDB structures were cleared of water molecules. The prepared 3D structures were further processed within the Chimera V 1.13.1 program. H5N1 Influenza construct, TLR7 and TLR8 have been then submitted to the HDOCK server to identify interaction regions. Taking into account the complete molecular interface between H5N1-TLRs, the complexes with the lowest intermolecular binding energy have been ranked best. The choice was made with the lowest mean Root Mean Square Deviation (RMSD). H-bond formation has been evaluated using LigPlot^+^. These steps describe the process of retrieving, preparing, and analyzing the 3D structures of the components and their interactions to comprehend the binding and molecular contacts among the H5N1 influenza construct and TLRs.

### Molecular dynamics and MM/PBSA analysis

Molecular dynamics simulations are a powerful tool for understanding the dynamic behavior of biomolecular complexes and are commonly used to investigate the interactions and stability of such complexes over time. The use of Gromacs and the CHARMM36 force field in this context helps provide valuable insights into the function of vaccine complexes in a simulated environment. Molecular dynamics calculations for the vaccine-m826 and vaccine-TLR7/8 complexes have been carried out using Gromacs v4.6.5 and the CHARMM36 all-atom force field. The protein–protein complexes have been solvated by adding water molecules, as well as the entire system has been then neutralized to ensure that it had an overall neutral charge. An energy minimization step was performed to optimize the geometry of the system and remove any steric clashes or unfavorable interactions. This helps to stabilize the system before proceeding to simulations. Optimization of the system was done for both NPT (constant number of particles, temperature and pressure) and NVT (constant number of particles, temperature and volume). These optimizations were carried out at 300 K (Kelvin), a 1 bar pressure, and with restraint forces set to 1000 kJ/mol. Following the optimization steps, molecular dynamics simulations were conducted for a total of 40,000 picoseconds (40 ns). During this simulation, the positions of all atoms in the system were updated over time to study the dynamics and behavior of the H5N1-TLR7 and H5N1-TLR8 complexes. To maintain stability during the simulation, all bonds have been constrained utilizing the LINCS (LINear Constraint Solver) algorithm, ensuring that bond lengths between atoms remain fixed.

### Simulations of H5N1 immune response

The C-ImmSim server was used to model and forecast the immunological response to recombinant influenza structures. To anticipate immunological interactions, C-ImmSim combines an agent-based model with machine learning methods and immune epitope prediction. It does this by using a position-specific scoring matrix (PSSM). C-ImmSim replicates three different mammalian anatomical areas at the same time: the bone marrow (representing hematopoietic stem cells, lymphoid cells, and myeloid cells), the thymus (mimicking native T cell selection), as well as a third lymph node resembling typical lymph nodes. The Simulation Steps parameter has been set to 1050 to account for the vaccine's 4-week gap between doses one and two. Time-steps of 1, 336, and 672, each corresponding to eight hours in real life, have been considered for the three injections. Different parameters used in this study were applied as default settings.

### Insilico cloning and vaccine optimization

The GeneScript program was used to optimize codons for the production of a recombinant construct in the pET-26b(+) vector. Because the expression host used in the investigation was different from the natural host for influenza, this optimization was required. To adapt the codon usage for the new host, based on the recommended codon use of influenza for production in Escherichia coli K12, the codon optimization has been modified. This ensures that the gene sequence is better suited for efficient expression in the new host. Two important metrics for evaluating codon optimization are the Codon Adaptation Index (CAI) score as well as the GC content. An ideal CAI score is 1.0, indicating a high level of adaptation to the host's codon usage, while a GC content of 30–70% is typically considered optimal for predicting protein expression levels in the host. To facilitate the cloning process, the locations of the *Nde*I as well as *Xho*I restriction enzyme sites have been recognized in the optimized nucleotide sequences. The insertion of the recombinant influenza gene sequence into the pET-26b(+) vector and effective target protein production in the selected host are made possible by these restriction sites. SnapGene has been utilized as a tool to aid in this identification and cloning process.

## Results

### Linear and conformational B cell epitope prediction

B cell epitopes have a crucial part in the H5N1 vaccine due to their ability to stimulate humoral immunity. To identify these epitopes, within the final design, both discontinuous and linear B cell epitopes have been predicted. Linear B cell epitopes have been determined utilizing BepiPred 2.0, while discontinuous epitopes were predicted with the IEDB servers. In total, 14 and 10 linear B cell epitopes have been detected for HA and NA, respectively, and detailed in Table 1. Additionally, Additional file 1: Tables S1 and S2 provide a summary of all B cell epitopes found within the NA and HA proteins.

**Table 1:** Liner B-cell epitopes predicted from NA and HA antigens

No	Start	End	IEDB	BepiPred 2.0
*HA protein*				
2	40	70	VTVTHAQDILEKTHNGKLCDLDGVKPLILRD	VTVTHAQDILEKTHNGKLCDLDGVKPLILRD
3	82	90	MCDEFINVP	MCDEFINVP
4	100	156	NPNNDLCYPGSFNDYEELKHLLSRINHFEKIQIIPKNSWSDHEASSGVSAACPYLGS	NPNNDLCYPGSFNDYEELKHLLSRINHFEKIQIIPKNSWSDHEASSGVSAACPYLGS
6	171	185	STYPTIKKSYNNTNQ	STYPTIKKSYNNTNQ
7	200	211	AAEQTRLYQNPT	AAEQTRLYQNPT
8	223	240	QRLVPKIATRSKVNGQSG	QRLVPKIATRSKVNGQSG
9	274	296	KKGDSAIMKSELEYGNCNTKCQT	KKGDSAIMKSELEYGNCNTKCQT
10	299	310	GAINSSMPFHNI	GAINSSMPFHN
11	330	346	ATGLRNSPQRESRRKKR	ATGLRNSPQRESRRKKR
12	359	397	GWQGMVDGWYGYHHSNEQGSGYAADKESTQKAVDGVTNK	GWQGMVDGWYGYHHSNEQGSGYAADKESTQKAVDGVTNK
16	410	420	EAVGREFNNLE	EAVGREFNNLE
18	452	460	RTLDFHDSN	RTLDFHDSN
20	476	481	AKELGN	AKELGN
21	495	527	MESIRNGTYNYPQYSEEARLKREEINGVKLESV	MESIRNGTYNYPQYSEEARLKREEINGVKLESV
*NA protein*				
1	38	55	IQKGNQHQAESISNTNPL	QKGNQHQAESISNTNPL
2	68	74	NSSLCPI	NSSLCPI
3	119	134	LMNDKHSNGTVKDRSP	LMNDKHSNGTVKDRSP
5	193	206	TDTIKSWRNNILRT	TDTIKSWRNNILRT
6	226	236	PSNGQASYKIF	PSNGQASYKIF
7	240	252	KGKVVKSVELDAP	KGKVVKSVELDAP
9	303	323	GDNPRPNDGTGSCGPMSPNGA	GDNPRPNDGTGSCGPMSPNGA
12	342	370	TKSTNSRSGFEMIWDPNGWTGTDSSFSVK	TKSTNSRSGFEMIWDPNGWTGTDSSFSVK
13	376	381	ITDWSG	ITDWSG
17	431	445	DTVSWSWPDGAELPF	DTVSWSWPDGAE

### Selection of CTL epitopes prediction

To predict CTL (Cytotoxic T Lymphocyte) epitopes, the NetMHC4 server has been employed specifically for the NA and HA proteins. The selection of MHC class I epitopes was based on their high levels of immunogenicity and antigenicity. Additionally, epitopes with a strong binding score, as determined by the NetMHC4 server, were considered. Select MHC-I epitopes were merged when they overlapped with one another. Ultimately, a total of 21 MHC class I epitopes were chosen, comprising 9 from HA and 12 from NA, which were integrated into the influenza vaccine construct (as detailed in Table 2). There are all predicted CTL epitopes, those that bind to MHC-I, within Additional file 1: Tables S3 and S4.

**Table 2:** MHC-I epitopes prediction. HA and NA epitopes have been chosen according to MHC-I HLAs binding affinity (nM), %rank and antigenicity rate

pos	HLA	Peptide	Affinity(nM)	Allergenicity	Toxicity	Solubility	Antigenicity
*HA protein*							
14	HLA-A0101	KSDQICIGY	38.08	No	Non-Toxin	Good	2.07
223	HLA-A3101	RLVPKIATR	9.27	No	Non-Toxin	Good	1.009
240	HLA-A3201	RMEFFWTIL	7.29	No	Non-Toxin	Good	1.195
304	HLA-A3207	MPFHNIHPL	17.58	No	Non-Toxin	Good	1.263
304	HLA-A3215	MPFHNIHPL	26.87	No	Non-Toxin	Good	1.263
304	HLA-A6601	MPFHNIHPL	986.12	No	Non-Toxin	Good	1.2633
304	HLA-A6823	MPFHNIHPL	9.68	No	Non-Toxin	Good	1.2633
304	HLA-B0801	MPFHNIHPL	40.2	No	Non-Toxin	Good	1.2633
304	HLA-B1402	MPFHNIHPL	980.92	No	Non-Toxin	Good	1.2633
239	HLA-B2705	GRMEFFWTI	16.64	No	Non-Toxin	Good	1.2086
239	HLA-B2720	GRMEFFWTI	8.67	No	Non-Toxin	Good	1.2086
304	HLA-B3501	MPFHNIHPL	6.01	No	Non-Toxin	Good	1.2633
150	HLA-B3503	CPYLGSPSF	1547.44	No	Non-Toxin	Good	1.1181
306	HLA-B3801	FHNIHPLTI	370.07	No	Non-Toxin	Good	1.3066
304	HLA-B3901	MPFHNIHPL	6.61	No	Non-Toxin	Good	1.2633
240	HLA-B4013	RMEFFWTIL	140.81	No	Non-Toxin	Good	1.1954
304	HLA-B4201	MPFHNIHPL	16.2	No	Non-Toxin	Good	1.2633
447	HLA-B4402	MENERTLDF	12.92	No	Non-Toxin	Good	1.5311
447	HLA-B4403	MENERTLDF	32.05	No	Non-Toxin	Good	1.5311
240	HLA-B4801	RMEFFWTIL	244.69	No	Non-Toxin	Good	1.1954
150	HLA-B5101	CPYLGSPSF	429.32	No	Non-Toxin	Good	1.1181
150	HLA-B5301	CPYLGSPSF	22.92	No	Non-Toxin	Good	1.1181
304	HLA-B5401	MPFHNIHPL	16.45	No	Non-Toxin	Good	1.2633
237	HLA-B5801	QSGRMEFFW	3.97	No	Non-Toxin	Good	1.0528
237	HLA-B5802	QSGRMEFFW	9413.88	No	Non-Toxin	Good	1.0528
304	HLA-B8301	MPFHNIHPL	97.78	No	Non-Toxin	Good	1.2633
*NA protein*							
105	HLA-A0202	HLECRIFFL	25.01	No	Non-Toxin	Good	1.1819
23	HLA-A0206	LQIGNIISI	7.02	No	Non-Toxin	Good	1.2621
393	HLA-A0207	GLDCIRPCF	13,931.8	No	Non-Toxin	Good	1.4833
7	HLA-A0211	ITIGSICMV	3.12	No	Non-Toxin	Good	2.0061
23	HLA-A0212	LQIGNIISI	10.88	No	Non-Toxin	Good	1.2621
105	HLA-A0217	HLECRIFFL	71.01	No	Non-Toxin	Good	1.1819
322	HLA-A2301	AYGVKGFSF	99.25	No	Non-Toxin	Good	1.2975
322	HLA-A2402	AYGVKGFSF	60.32	No	Non-Toxin	Good	1.2975
14	HLA-A2601	MVIGMVSLM	5.23	No	Non-Toxin	Good	1.0720
14	HLA-A2602	MVIGMVSLM	6.85	No	Non-Toxin	Good	1.0720
324	HLA-A2902	GVKGFSFKY	22.73	No	Non-Toxin	Good	1.1111
324	HLA-A3002	GVKGFSFKY	67.34	No	Non-Toxin	Good	1.1111
14	HLA-A6601	MVIGMVSLM	2301.39	No	Non-Toxin	Good	1.0720
105	HLA-B0802	HLECRIFFL	1975.27	No	Non-Toxin	Good	1.1819
105	HLA-B0803	HLECRIFFL	5079.26	No	Non-Toxin	Good	1.1819
291	HLA-B1501	YQIGYICSG	66.4	No	Non-Toxin	Good	1.3358
289	HLA-B4001	LEYQIGYIC	158.53	No	Non-Toxin	Good	1.9916
289	HLA-B4002	LEYQIGYIC	41.21	No	Non-Toxin	Good	1.9916
13	HLA-B4801	CMVIGMVSL	1001.31	No	Non-Toxin	Good	1.9406
397	HLA-C0702	IRPCFWVEL	644.03	No	Non-Toxin	Good	1.1888
328	HLA-C0802	FSFKYGNGV	891.48	No	Non-Toxin	Good	1.3196
328	HLA-C1203	FSFKYGNGV	12.1	No	Non-Toxin	Good	1.3196

### Selection of HTL epitopes prediction

For the NA and HA antigens, the method used to predict how peptides will attach to MHC class II alleles has been the NetMHCII-2.3 server. In this process, all MHC class II epitopes have been thoroughly examined, and the epitopes have been chosen according to their strong immunogenicity, and antigenicity, as well as the highest binding score. Additionally, independent MHC class II epitopes were considered when they exhibited overlapping regions. Ultimately, an overall of 9 MHC class II epitopes have been selected for the construction of the H5N1 Vaccine, consisting of 6 HA epitopes and 3 NA epitopes, as outlined in Table 3. Thus, all the predicted HTL (Helper T Lymphocyte) epitopes, which bind to MHC class II, can be found in Additional file 1: Tables S5 and S6.

**Table 3:** MHC-II epitopes prediction from HA and NA proteins

Pos	Allele	Peptide	Affinity (nM)	Antigenicity
*HA protein*				
462–477	DRB1_0103	NLYDKVRLQLRDNAK	2778.9	1.1390
443–457	DRB1_0301	ELLVLMENERTLDFH	87.4	1.0452
210–224	DRB1_0401	PTTYISIGTSTLNQR	19.7	1.0883
442–456	DRB1_0405	AELLVLMENERTLDF	20.4	1.0504
464–476	DRB4_0101	LYDKVRLQLRDNAKE	25.6	1.0144
210–224	HLA-DQA10501-DQB10301	PTTYISIGTSTLNQR	29.9	1.0883
*NA protein*				
9–23	DRB1_0403	TIGSICMVIGMVSLM	0.1	1.4483
13–27	HLA-DQA10102-DQB10501	ICMVIGMVSLMLQIG	0.2	1.5671
10–24	HLA-DQA10103-DQB10603	GSICMVIGMVSLMLQ	0.5	1.2727

### Population coverage

**Fig. 1:**
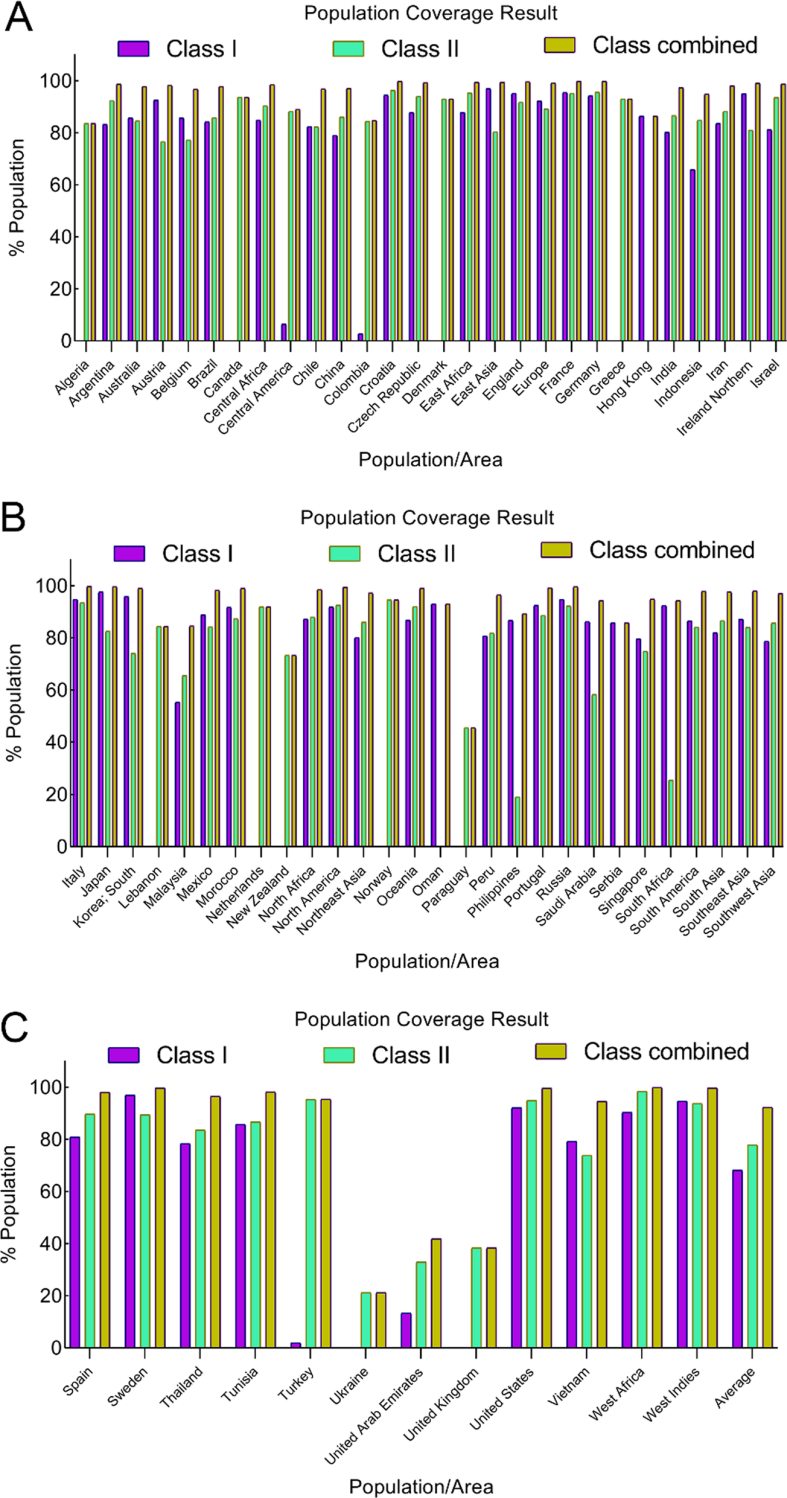
Population coverage (%) for specific epitopes containing HLA-binding alleles. For each chosen epitope, a global and average population coverage percentage is taken into account. The colors of MHC class I, and class II, as well as combined class I and class II are displayed in purple, green, and olive-green, correspondingly

The IEDB population coverage instrument, accessible at http://tools.iedb.org/population, has been employed to assess the extent of population coverage for each chosen epitope. As illustrated in Table 4, the greatest population coverage was found when both Class I and Class II epitopes were taken into account. This is noteworthy because NA and HA antigens linked to the [Influenza A virus (A/chicken/EastJava/UT551/2010(H5N1))] antigen originate in the Asian region. A comprehensive breakdown of population coverage in terms of Class I, and Class II, as well as the combined Class I and II epitopes associated with various continents has been presented in Table 4 and Fig. 1.

**Table 4:** Population coverage (%) for specific epitopes containing HLA-binding alleles of MHC class I, class II, as well as combined class I and class II

population/area	Class I			Class II			Class combined		
	Coverage^a^	Average_hit^b^	pc90^c^	Coverage^a^	Average_hit^b^	pc90^c^	Coverage^a^	Average_hit^b^	pc90^c^
Algeria	0.0%	0.0	0.0	83.55%	1.42	0.61	83.55%	1.42	0.61
Argentina	83.23%	1.73	0.6	92.37%	1.78	1.07	98.72%	3.51	2.03
Australia	85.67%	1.71	0.7	84.53%	1.31	0.65	97.78%	3.02	1.63
Austria	92.54%	2.28	1.09	76.61%	1.24	0.43	98.26%	3.52	1.85
Belgium	85.65%	2.06	0.7	77.25%	1.25	0.44	96.74%	3.3	1.52
Brazil	84.17%	1.74	0.63	85.76%	1.43	0.7	97.75%	3.18	1.69
Canada	0.0%	0.0	0.0	93.63%	1.66	1.11	93.63%	1.66	1.11
Central Africa	84.85%	1.6	0.66	90.38%	1.65	1.01	98.54%	3.24	1.96
Central America	6.44%	0.07	0.11	88.25%	1.39	0.85	89.0%	1.46	0.91
Chile	82.4%	1.64	0.57	82.25%	1.45	0.56	96.88%	3.09	1.52
China	78.98%	1.39	0.48	86.03%	1.5	0.72	97.06%	2.88	1.52
Colombia	2.84%	0.03	0.1	84.34%	1.45	0.64	84.79%	1.48	0.66
Croatia	94.5%	2.33	1.19	96.26%	2.13	1.27	99.79%	4.46	2.86
Czech Republic	87.79%	2.07	0.82	94.04%	2.05	1.15	99.27%	4.12	2.29
Denmark	0.0%	0.0	0.0	93.03%	2.29	1.13	93.03%	2.29	1.13
East Africa	87.8%	1.9	0.82	95.27%	1.96	1.2	99.42%	3.86	2.31
East Asia	96.97%	2.36	1.37	80.31%	1.37	0.51	99.4%	3.73	2.29
England	95.12%	2.74	1.24	91.7%	2.12	1.07	99.59%	4.86	2.74
Europe	92.13%	2.33	1.08	89.14%	1.82	0.92	99.15%	4.15	2.28
France	95.51%	2.63	1.28	95.14%	2.32	1.24	99.78%	4.94	3.04
Germany	94.18%	2.5	1.18	95.55%	2.27	1.25	99.74%	4.77	2.87
Greece	0.0%	0.0	0.0	92.99%	1.88	1.1	92.99%	1.88	1.1
Hong Kong	86.36%	1.59	0.73	0.0%	0.0	0.0	86.36%	1.59	0.73
India	80.13%	1.61	0.5	86.55%	1.58	0.74	97.33%	3.19	1.61
Indonesia	65.87%	1.02	0.29	84.82%	1.31	0.66	94.82%	2.33	1.22
Iran	83.59%	1.46	0.61	88.19%	1.62	0.85	98.06%	3.08	1.77
Ireland Northern	95.02%	2.69	1.23	81.01%	1.46	0.53	99.05%	4.15	2.21
Israel	81.19%	1.75	0.53	93.51%	1.94	1.13	98.78%	3.69	2.08
Italy	94.7%	2.53	1.2	93.46%	1.84	1.11	99.65%	4.37	2.58
Japan	97.56%	2.39	1.44	82.55%	1.4	0.57	99.57%	3.79	2.4
Korea; South	95.86%	2.26	1.27	74.06%	1.16	0.39	98.93%	3.42	2.08
Lebanon	0.0%	0.0	0.0	84.42%	1.38	0.64	84.42%	1.38	0.64
Malaysia	55.3%	0.78	0.22	65.45%	0.85	0.29	84.56%	1.63	0.65
Mexico	88.79%	1.78	0.89	84.16%	1.41	0.63	98.22%	3.19	1.83
Morocco	91.75%	2.32	1.07	87.3%	1.61	0.79	98.95%	3.93	2.19
Netherlands	0.0%	0.0	0.0	91.94%	2.0	1.07	91.94%	2.0	1.07
New Zealand	0.0%	0.0	0.0	73.31%	1.09	0.37	73.31%	1.09	0.37
North Africa	87.26%	1.9	0.78	87.97%	1.67	0.83	98.47%	3.56	2.02
North America	91.84%	2.14	1.07	92.53%	2.0	1.09	99.39%	4.13	2.39
Northeast Asia	80.08%	1.41	0.5	86.03%	1.5	0.72	97.22%	2.91	1.55
Norway	0.0%	0.0	0.0	94.51%	2.34	1.2	94.51%	2.34	1.2
Oceania	86.77%	1.74	0.76	91.99%	1.77	1.06	98.94%	3.5	2.09
Oman	93.04%	2.14	1.11	0.0%	0.0	0.0	93.04%	2.14	1.11
Paraguay	0.0%	0.0	0.0	45.49%	0.53	0.18	45.49%	0.53	0.18
Peru	80.75%	1.72	0.52	81.81%	1.31	0.55	96.5%	3.03	1.44
Philippines	86.69%	1.73	0.75	19.14%	0.2	0.12	89.24%	1.92	0.93
Portugal	92.4%	2.16	1.09	88.6%	1.73	0.88	99.13%	3.89	2.24
Russia	94.7%	2.45	1.21	92.16%	1.99	1.08	99.58%	4.44	2.6
Saudi Arabia	86.23%	1.96	0.73	58.23%	0.8	0.24	94.25%	2.76	1.23
Serbia	85.71%	1.2	0.7	0.0%	0.0	0.0	85.71%	1.2	0.7
Singapore	79.61%	1.4	0.49	74.83%	0.92	0.4	94.87%	2.31	1.22
South Africa	92.34%	2.14	1.09	25.52%	0.26	0.13	94.3%	2.4	1.2
South America	86.48%	1.71	0.74	84.11%	1.42	0.63	97.85%	3.13	1.7
South Asia	81.86%	1.66	0.55	86.6%	1.59	0.75	97.57%	3.24	1.67
Southeast Asia	87.1%	1.66	0.77	84.03%	1.43	0.63	97.94%	3.1	1.74
Southwest Asia	78.69%	1.58	0.47	85.65%	1.53	0.7	96.94%	3.1	1.53
Spain	80.9%	1.37	0.52	89.68%	1.78	0.97	98.03%	3.15	1.77
Sweden	96.98%	2.44	1.33	89.41%	1.85	0.94	99.68%	4.3	2.57
Thailand	78.36%	1.36	0.46	83.55%	1.42	0.61	96.44%	2.78	1.43
Tunisia	85.74%	1.91	0.7	86.64%	1.66	0.75	98.09%	3.57	1.9
Turkey	1.99%	0.02	0.1	95.32%	2.07	1.21	95.41%	2.09	1.22
Ukraine	0.0%	0.0	0.0	21.22%	0.42	0.25	21.22%	0.42	0.25
United Arab Emirates	13.32%	0.14	0.12	32.92%	0.33	0.15	41.86%	0.47	0.17
United Kingdom	0.0%	0.0	0.0	38.38%	0.38	0.16	38.38%	0.38	0.16
United States	92.06%	2.15	1.07	94.95%	2.13	1.19	99.6%	4.29	2.54
Vietnam	79.28%	1.42	0.48	73.86%	0.97	0.38	94.58%	2.39	1.21
West Africa	90.38%	1.88	1.01	98.42%	2.36	1.55	99.85%	4.24	2.85
West Indies	94.69%	2.35	1.21	93.76%	1.93	1.13	99.67%	4.28	2.65
Average	68.12	1.49	0.66	77.89	1.45	0.73	92.24	2.94	1.63
Standard deviation	36.06	0.87	0.43	24.34	0.6	0.37	14.94	1.15	0.74

^a^Projected population coverage

^b^Average number of epitope hits / HLA combinations recognized by the population

^c^Minimum number of epitope hits / HLA combinations recognized by 90% of the population

### Conservancy selected epitopes

The IEDB conservancy analysis instrument has been used to be a monitoring instrument for examining the level of conservation in chosen epitopes relative to other similar sequences. As shown in Table 5 epitopes exhibit varying lengths of minimum 0 and maximum 100 percent conservation.

**Table 5:** Conservancy selected epitopes from HA and NA epitopes

Allele	Epitope sequence	Epitope length	Percent of protein sequence m atches at identity < = 100%	Minim um identity (%)	Maxim um identity (%)
*HA Protein*					
HLA-A0101	KSDQICIGY	9	99.00% (99/100)	88.89	100.00
HLA-A3101	RLVPKIATR	9	82.00% (82/100)	66.67	100.00
HLA-A3201	RMEFFWTIL	9	96.00% (96/100)	88.89	100.00
HLA-A3207	MPFHNIHPL	9	100.00% (100/100)	100.00	100.00
HLA-A3215	MPFHNIHPL	9	100.00% (100/100)	100.00	100.00
HLA-A6601	MPFHNIHPL	9	100.00% (100/100)	100.00	100.00
HLA-A6823	MPFHNIHPL	9	100.00% (100/100)	100.00	100.00
HLA-B0801	MPFHNIHPL	9	100.00% (100/100)	100.00	100.00
HLA-B1402	MPFHNIHPL	9	100.00% (100/100)	100.00	100.00
HLA-B2705	GRMEFFWTI	9	95.00% (95/100)	88.89	100.00
HLA-B2720	GRMEFFWTI	9	95.00% (95/100)	88.89	100.00
HLA-B3501	MPFHNIHPL	9	100.00% (100/100)	100.00	100.00
HLA-B3503	CPYLGSPSF	9	88.00% (88/100)	77.78	100.00
HLA-B3801	FHNIHPLTI	9	99.00% (99/100)	88.89	100.00
HLA-B3901	MPFHNIHPL	9	100.00% (100/100)	100.00	100.00
HLA-B4013	RMEFFWTIL	9	96.00% (96/100)	88.89	100.00
HLA-B4201	MPFHNIHPL	9	100.00% (100/100)	100.00	100.00
HLA-B4402	MENERTLDF	9	100.00% (100/100)	100.00	100.00
HLA-B4403	MENERTLDF	9	100.00% (100/100)	100.00	100.00
HLA-B4801	RMEFFWTIL	9	96.00% (96/100)	88.89	100.00
HLA-B5101	CPYLGSPSF	9	88.00% (88/100)	77.78	100.00
HLA-B5301	CPYLGSPSF	9	88.00% (88/100)	77.78	100.00
HLA-B5401	MPFHNIHPL	9	100.00% (100/100)	100.00	100.00
HLA-B5801	QSGRMEFFW	9	99.00% (99/100)	77.78	100.00
HLA-B5802	QSGRMEFFW	9	99.00% (99/100)	77.78	100.00
HLA-B8301	MPFHNIHPL	9	100.00% (100/100)	100.00	100.00
DRB1_0103	NLYDKVRLQLRDNAK	15	96.00% (96/100)	93.33	100.00
DRB1_0301	ELLVLMENERTLDFH	15	100.00% (100/100)	100.00	100.00
DRB1_0401	PTTYISIGTSTLNQR	15	83.00% (83/100)	93.33	100.00
DRB1_0405	AELLVLMENERTLDF	15	100.00% (100/100)	100.00	100.00
DRB4_0101	LYDKVRLQLRDNAKE	15	96.00% (96/100)	93.33	100.00
HLA-DQA10501-DQB10301	PTTYISIGTSTLNQR	15	83.00% (83/100)	93.33	100.00
*NA Protein*					
HLA-A0202	HLECRIFFL	9	2.00% (2/100)	88.89	100.00
HLA-A0206	LQIGNIISI	9	6.00% (6/100)	66.67	100.00
HLA-A0207	GLDCIRPCF	9	100.00% (100/100)	100.00	100.00
HLA-A0211	ITIGSICMV	9	95.00% (95/100)	88.89	100.00
HLA-A0212	LQIGNIISI	9	6.00% (6/100)	66.67	100.00
HLA-A0217	HLECRIFFL	9	2.00% (2/100)	88.89	100.00
HLA-A2301	AYGVKGFSF	9	100.00% (100/100)	100.00	100.00
HLA-A2402	AYGVKGFSF	9	100.00% (100/100)	100.00	100.00
HLA-A2601	MVIGMVSLM	9	2.00% (2/100)	77.78	100.00
HLA-A2602	MVIGMVSLM	9	2.00% (2/100)	77.78	100.00
HLA-A2902	GVKGFSFKY	9	100.00% (100/100)	100.00	100.00
HLA-A3002	GVKGFSFKY	9	100.00% (100/100)	100.00	100.00
HLA-A6601	MVIGMVSLM	9	2.00% (2/100)	77.78	100.00
HLA-B0802	HLECRIFFL	9	2.00% (2/100)	88.89	100.00
HLA-B0803	HLECRIFFL	9	2.00% (2/100)	88.89	100.00
HLA-B1501	YQIGYICSG	9	99.00% (99/100)	88.89	100.00
HLA-B4001	LEYQIGYIC	9	99.00% (99/100)	88.89	100.00
HLA-B4002	LEYQIGYIC	9	99.00% (99/100)	88.89	100.00
HLA-B4801	CMVIGMVSL	9	2.00% (2/100)	77.78	100.00
HLA-C0702	IRPCFWVEL	9	100.00% (100/100)	100.00	100.00
HLA-C0802	FSFKYGNGV	9	100.00% (100/100)	100.00	100.00
HLA-C1203	FSFKYGNGV	9	100.00% (100/100)	100.00	100.00
DRB1_0403	TIGSICMVIGMVSLM	15	2.00% (2/100)	86.67	100.00
HLA-DQA10102-DQB10501	ICMVIGMVSLMLQIG	15	2.00% (2/100)	86.67	100.00
HLA-DQA10103-DQB10603	GSICMVIGMVSLMLQ	15	2.00% (2/100)	86.67	100.00

### Final epitopes selection, construction design and physicochemical properties

The nucleotide sequence of H5N1 can be found in Additional file 1: Table S7. Final selected epitopes and adjuvants are presented in Table 6.

**Table 6:** Final selected epitopes. All overlapping sequences and epitopes are labeled as underlined and in bold form

Epitopes/Adjuvants	Type
MRTLDFHDSN	B Cell
NSSLCPIK	B Cell
SDQICIGY	MHC-I
CPYLGSPSF	MHC-I
RLVPKIATR	MHC-I
QSGRMEFFWL	MHC-I
MPFHNIHPLTI	MHC-I
ITIGSICMV	MHC-I
MVIGMVSLM	MHC-I
LQIGNIISI	MHC-I
HLECRIFFL	MHC-I
LEYQIGYICSG	MHC-I
AYGVKGFSFKY	MHC-I
GLDCIRPCF	MHC-I
PTTYISIGTSTLNQR	MHC-II
AELLVLMENERTLDFH	MHC-II
NLYDKVRLQLRDNAKE	MHC-II
MRIHYLLFALLFLFLVPV	
PGHGGIINTLQKYYCRVR	
GGRCAVLSCLPKEEQIGK	
CSTRGRKCCRRKK	Adjuvant
AKFVAAWTLKAAA	Adjuvant

**Fig. 2:**
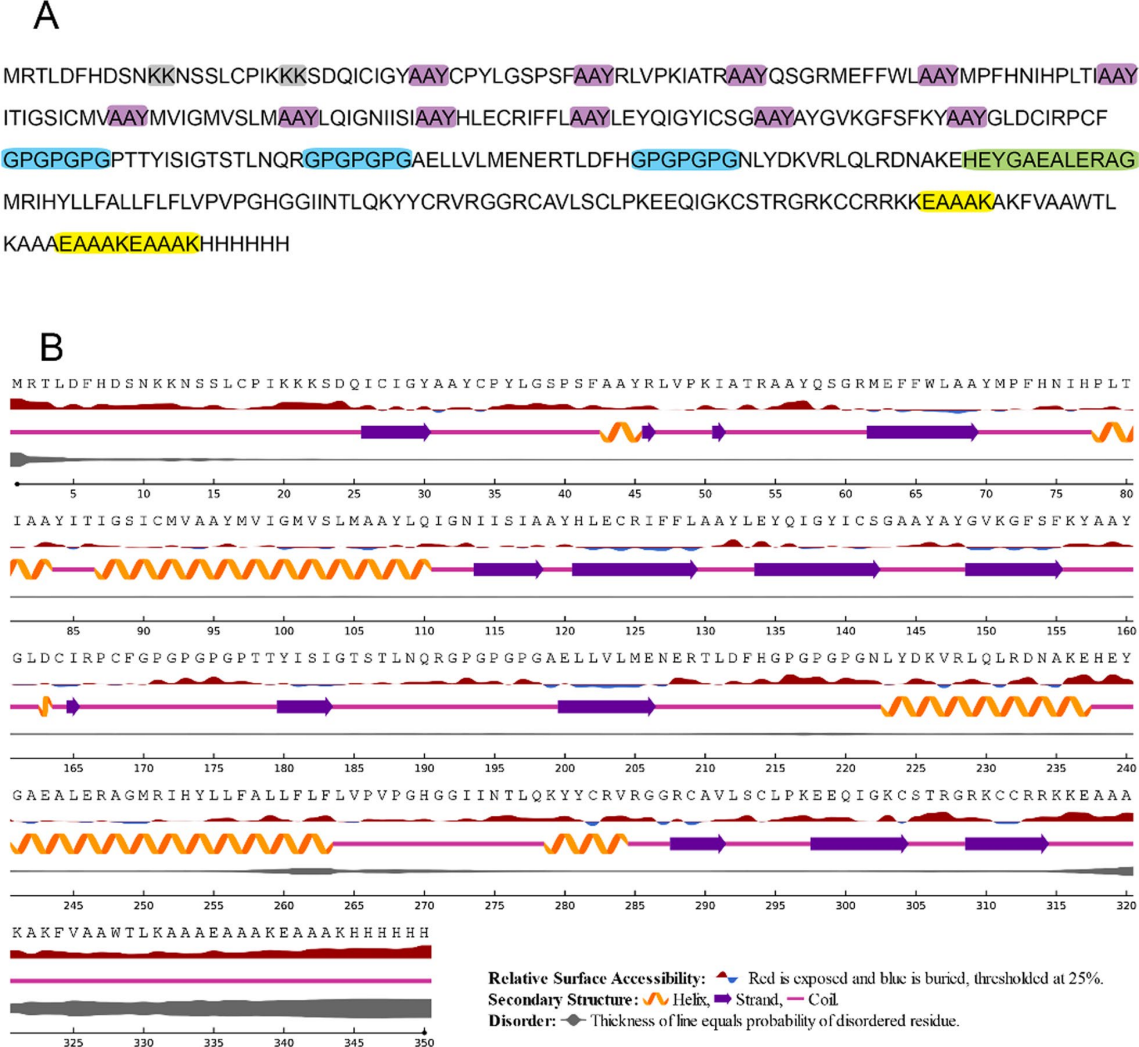
**A** Amino acid sequence of H5N1 construct. Amino acids in gray are KK linkers, Amino acids in purple are AAY linkers, amino acids in blue are GPGPGPG linkers, amino acids in green are HEYGAEALERAG and amino acids in yellow are EAAAK linkers. **B** Secondary structure of H5N1 construct. Helixes are shown as orange color, Beta strands are shown as purple color and coils are shown as pink color and disorders are shown as grey color

The final selection of epitopes was interconnected using specific linkers. AAY linkers were chosen to link the MHC-I epitopes, and GPGPGPG linkers have been used for linking the MHC-II epitopes. Adjuvants such as HβD-3 and the PADRE sequence have been connected using EAAAK linkers at the C-terminal site. To bridge the final MHC-I epitope to the initial MHC-II epitope, the HEYGAEALERAG linker was employed. KK linkers were utilized for connecting B-cell epitopes. To complete the structure, a "Histidine Tag" has been included at the C-terminal and attached to the last construct as depicted in Fig. 2A. Also, the secondary structure of the H5N1 construct is shown in Fig. 2B.

The outcomes obtained from the ProtParam server revealed that the H5N1 vaccine amino acid sequence, which consists of 350 amino acids, is expected to produce a protein with a molecular weight of ~ 38.46 kDa. The theoretical isoelectric point (pI) and instability index (II) have been determined at 9.32 and 34.44, respectively, indicating that the influenza vaccine falls within the category of stable proteins. Moreover, this vaccine's half-life in a variety of prokaryotic and eukaryotic hosts is predicted to be between 10 and 30 h. The aliphatic index is 84.37 and the grand average of hydropathicity (GRAVY) to be 0.018.

### Discontinues B cell from final H5N1 construct

**Fig. 3:**
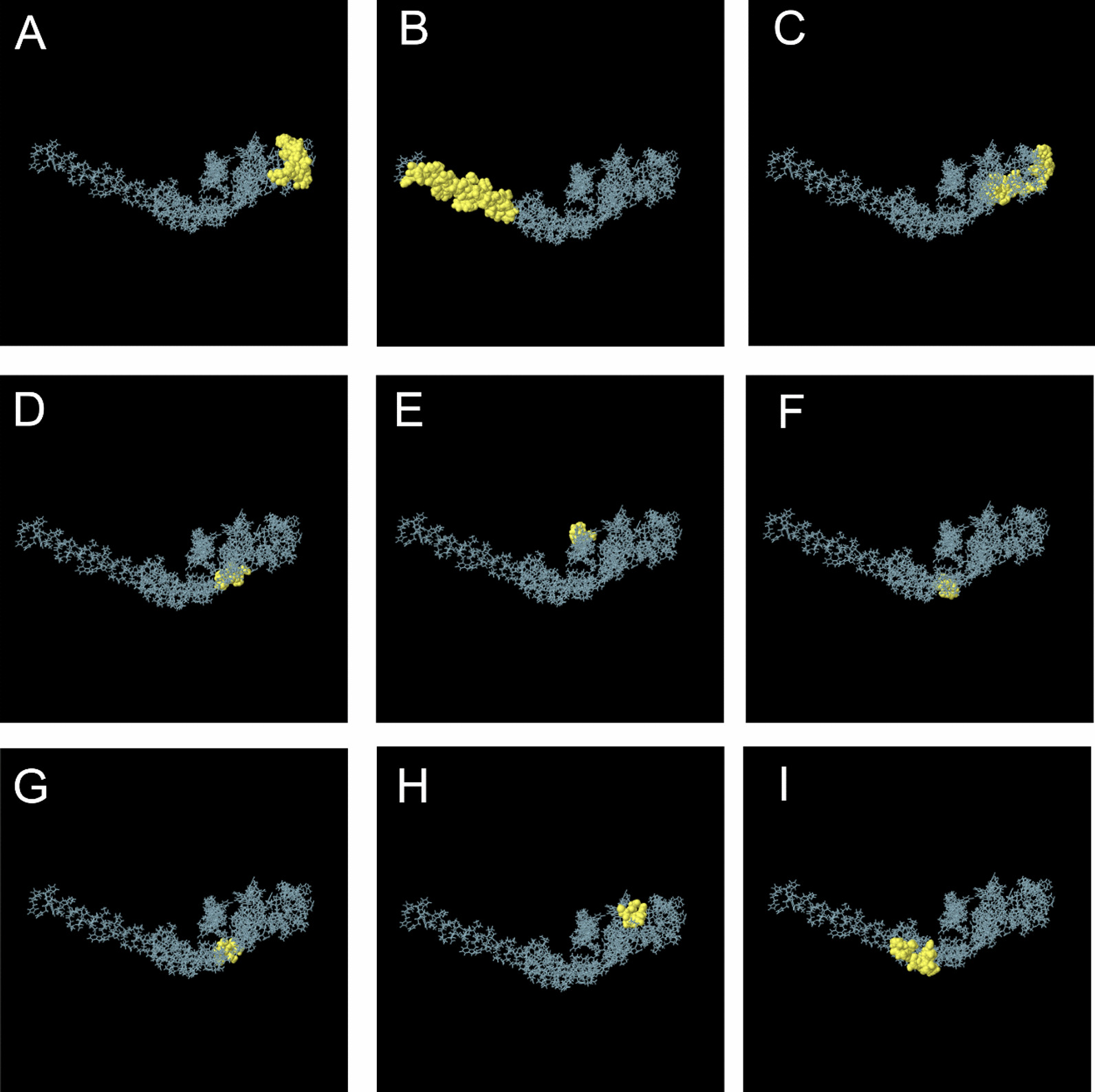
3D visualization of discontinuous B cell epitopes in H5N1 construct. Yellow regions are related to discontinuous amino acids in the final H5N1 construct. **A** Amino acids of F169, G170, P171, G172, P173, G174, P175, G176, P177, T178, T179, Y180, I181, S182, I183, G184, T185, S186, T187, L188, N189, Q190 and R191. **B** Amino acids of K315, K316, E317, A318, A319, A320, K321, A322, K323, F324, V325, A326, A327, W328, T329, L330, K331, A332, A333, A334, E335, A336, A337, A338, K339, E340, A341, A342, A343, K344, H346 and H347. **C** Amino acids of R209, T210, L211, D212, F213, H214, G215, P216, G217, P218, G219, P220, G221, N222, L223, Y224, D225, K226, V227, R228, L229, Q230, L231 and R232. **D**Amino acids of D233, N234, A235, K236, E237, E239, and Y240. **E** Amino acids of P78, L79, T80, I81, A82 and I85. **F** Amino acids of M250, R251, and Y254. **G** Amino acids of G241, A242, E243, A244, E246, and R247. **H** G161, L162, D163, C164, I165, and R166. (I)Amin acids of V291, L292, S293, C294, L295, P296, K297, S305, T306, R307, G308, and R309

Table 7 provides an overview of the conformational B cell epitopes from the final construct of the H5N1 vaccine. Each of the eight predicted scores for conformational B cell epitopes is shown in Fig. 3.

**Table 7:** Predicted discontinuous epitope of final H5N1 construct

No	Residues	Number of residues	Score
1	A:F169, A:G170, A:P171, A:G172, A:P173, A:G174, A:P175, A:G176, A:P177, A:T178, A:T179, A:Y180, A:I181, A:S182, A:I183, A:G184, A:T185, A:S186, A:T187, A:L188, A:N189, A:Q190, A:R191	23	0.813
2	A:K315, A:K316, A:E317, A:A318, A:A319, A:A320, A:K321, A:A322, A:K323, A:F324, A:V325, A:A326, A:A327, A:W328, A:T329, A:L330, A:K331, A:A332, A:A333, A:A334, A:E335, A:A336, A:A337, A:A338, A:K339, A:E340, A:A341, A:A342, A:A343, A:K344, A:H346, A:H347	32	0.791
3	A:R209, A:T210, A:L211, A:D212, A:F213, A:H214, A:G215, A:P216, A:G217, A:P218, A:G219, A:P220, A:G221, A:N222, A:L223, A:Y224, A:D225, A:K226, A:V227, A:R228, A:L229, A:Q230, A:L231, A:R232	24	0.777
4	A:D233, A:N234, A:A235, A:K236, A:E237, A:E239, A:Y240	7	0.7
5	A:P78, A:L79, A:T80, A:I81, A:A82, A:I85	6	0.695
6	A:M250, A:R251, A:Y254	3	0.656
7	A:G241, A:A242, A:E243, A:A244, A:E246, A:R247	6	0.638
8	A:G161, A:L162, A:D163, A:C164, A:I165, A:R166	6	0.615
9	A:V291, A:L292, A:S293, A:C294, A:L295, A:P296, A:K297, A:S305, A:T306, A:R307, A:G308, A:R309	12	0.566

### Modeling of 3D construct, refinement and validation

**Fig. 4:**
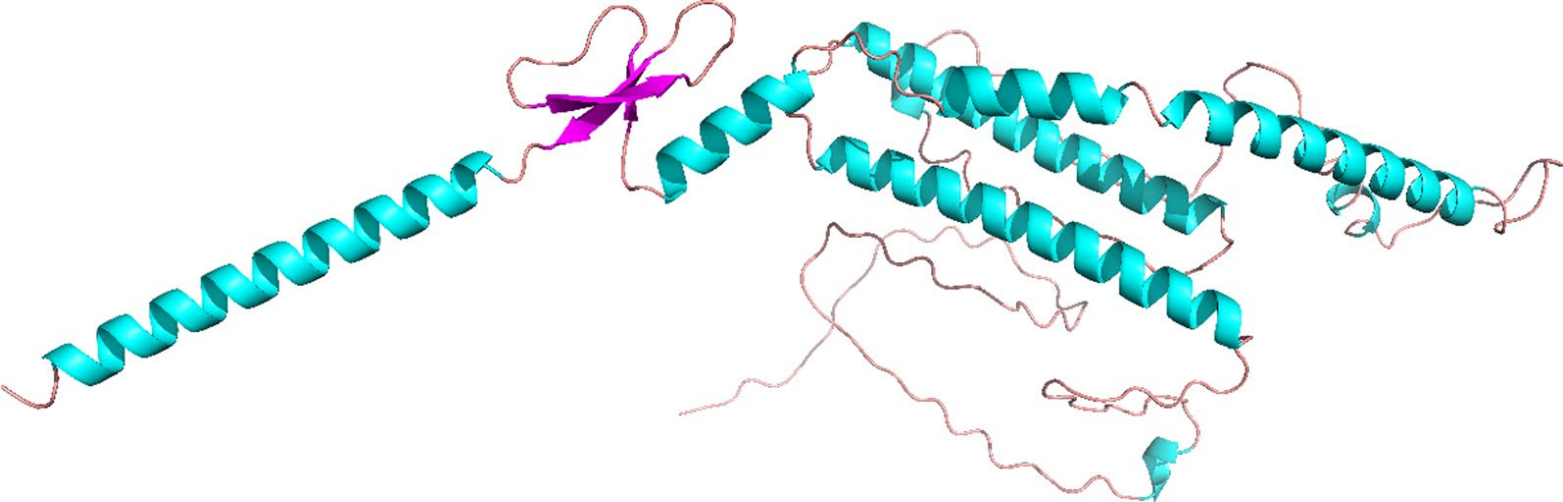
3D structure of H5N1. Coils are shown as brown, α-Helixes are shown as blue and β-Sheets are shown as purple

**Fig. 5:**
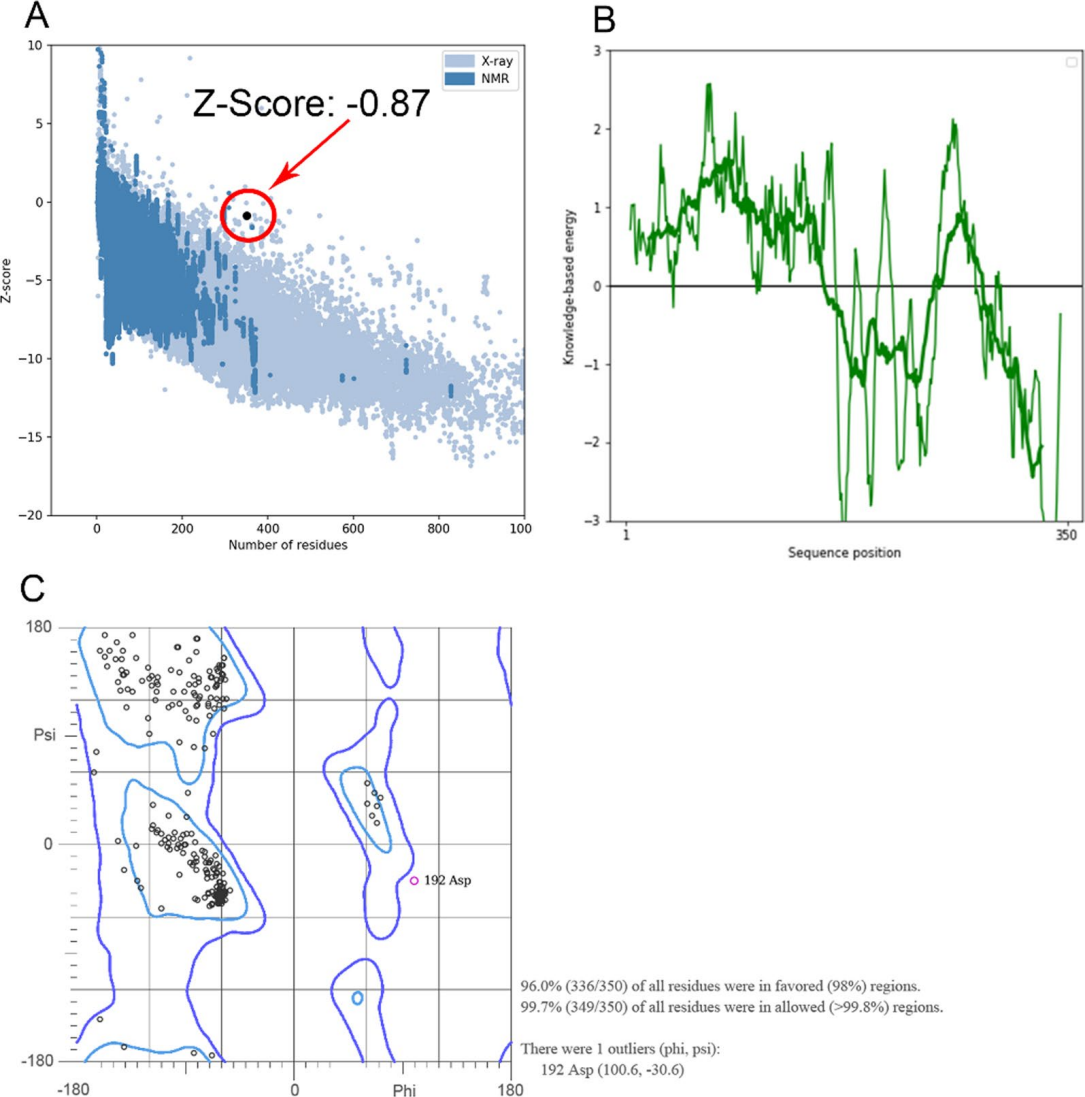
**A** Z-Score validation of H5N1 3D homology modeling. A value of − 0.87 shows that the 3D H5N1 structure is located in X-ray crystallographic proteins. **B** The local model quality of H5N1 confirms that the energy of this structure is close to a stable manner. **C** Ramachandran plot associated with vaccine 3D structure. 98% of residues are modeled in favored regions and more than 99.8% of residues are modeled in allowed regions

The 3D structure of H5N1 has been modeled using the RoseTTAFold server, as illustrated in Fig. 4. Subsequently, the obtained H5N1 3D structure was subjected to the GalaxyRefine server for further refinement. Model validation was carried out using online software PROCHECK and PROSA. After refining, the findings showed a Z-Score of -0.87, which is used to evaluate the quality of the modeled protein based on NMR or X-ray methodologies (Fig. 5A). For proteins with less than 200 amino acids, the NMR methodology is usually utilized, however for proteins with more than 200 amino acids, the X-ray methodology is applied. The (blue-blue) NMR and (pale-blue) X-ray zones on the PROSA graph denote great simulation accuracy, the lowest error rate, as well as the strongest confidence in the simulated model, respectively, if the Z-Score dot falls inside them. Local model quality associated with the protein's structure is depicted in Fig. 5B.

Based on the Ramachandran graph analysis of the anticipated model, 336 out of 350 residues, or 96% of all residues, were found inside the preferred areas (with a 98% probability). In total, approximately 99.7% of all residues, or 349 out of 350, were within the allowed regions (with a likelihood exceeding 99.8%). The Ramachandran graph organizes amino acids based on the angles of phi and psi, as shown in Fig. 5C. This data reflects the overall quality and reliability of the model.

### Protein–protein docking

The structures of H5N1 including TLR7 and TLR8 proteins have been submitted to the HDOCK server to determine the contact areas by protein–protein docking. The highest-ranking results for every complex have been chosen based on the least intermolecular binding energy among entire H5N1-Proteins complexes with the lowest docking score as shown in Tables 8 and 9.

**Table 8:** An overview of the top ten H5N1-TLR7 models

Rank	1	2	3	4	5	6	7	8	9	10
Docking Score	− 374.08	− 373.18	− 366.36	− 361.21	− 358.82	− 357.58	− 357.20	− 349.83	− 349.25	− 341.88
Confidence Score	0.9888	0.9886	0.9870	0.9856	0.9849	0.9845	0.9844	0.9820	0.9817	0.9789
Ligand rmsd (Å)	92.47	70.15	71.18	68.52	80.36	122.48	69.70	54.50	68.29	88.55
Interface residues	Model_1	Model_2	Model_3	Model_4	Model_5	Model_6	Model_7	Model_8	Model_9	Model_10

**Table 9:** An overview of the top ten H5N1-TLR8

Rank	1	2	3	4	5	6	7	8	9	10
Docking Score	− 414.39	− 371.33	− 351.41	− 335.02	− 331.52	− 328.21	− 318.14	− 316.39	− 313.14	− 311.12
Confidence Score	0.9950	0.9882	0.9825	0.9759	0.9742	0.9725	0.9665	0.9654	0.9631	0.9617
Ligand rmsd (Å)	91.61	93.05	64.85	57.21	58.70	80.88	76.69	92.16	87.30	70.86
Interface residues	Model_1	Model_2	Model_3	Model_4	Model_5	Model_6	Model_7	Model_8	Model_9	Model_10

**Fig. 6:**
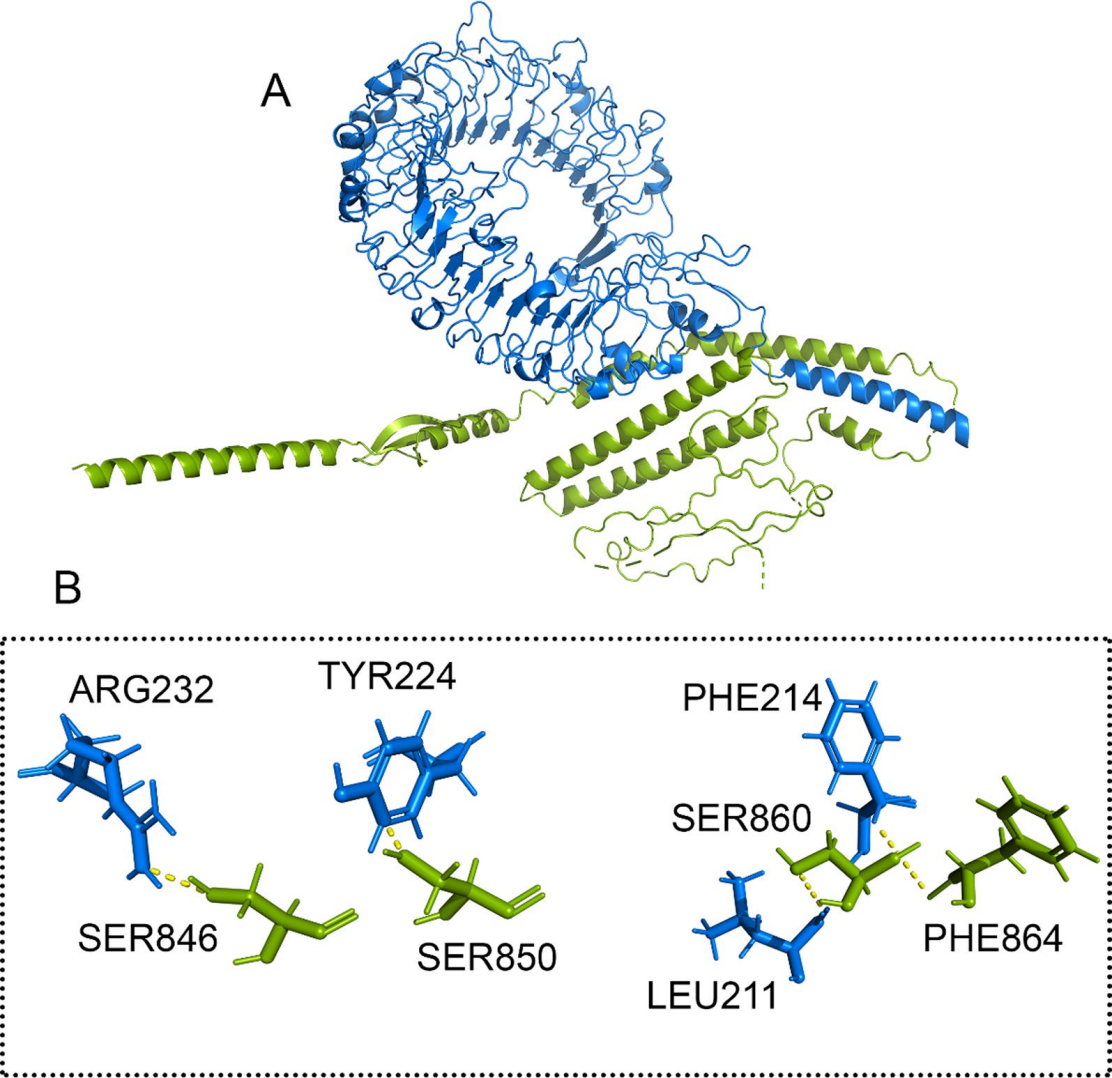
**A** Docking HADDOCK outcomes for the H5N1-TLR7 complex are visualized, **B** and the residues' interaction with the complex has been magnified. The H5N1 structure is shown as a green stick-filled carton. The blue cartons and sticks that make up the TLR7 structure are displayed. The dashed line in yellow represents hydrogen bonds

**Fig. 7:**
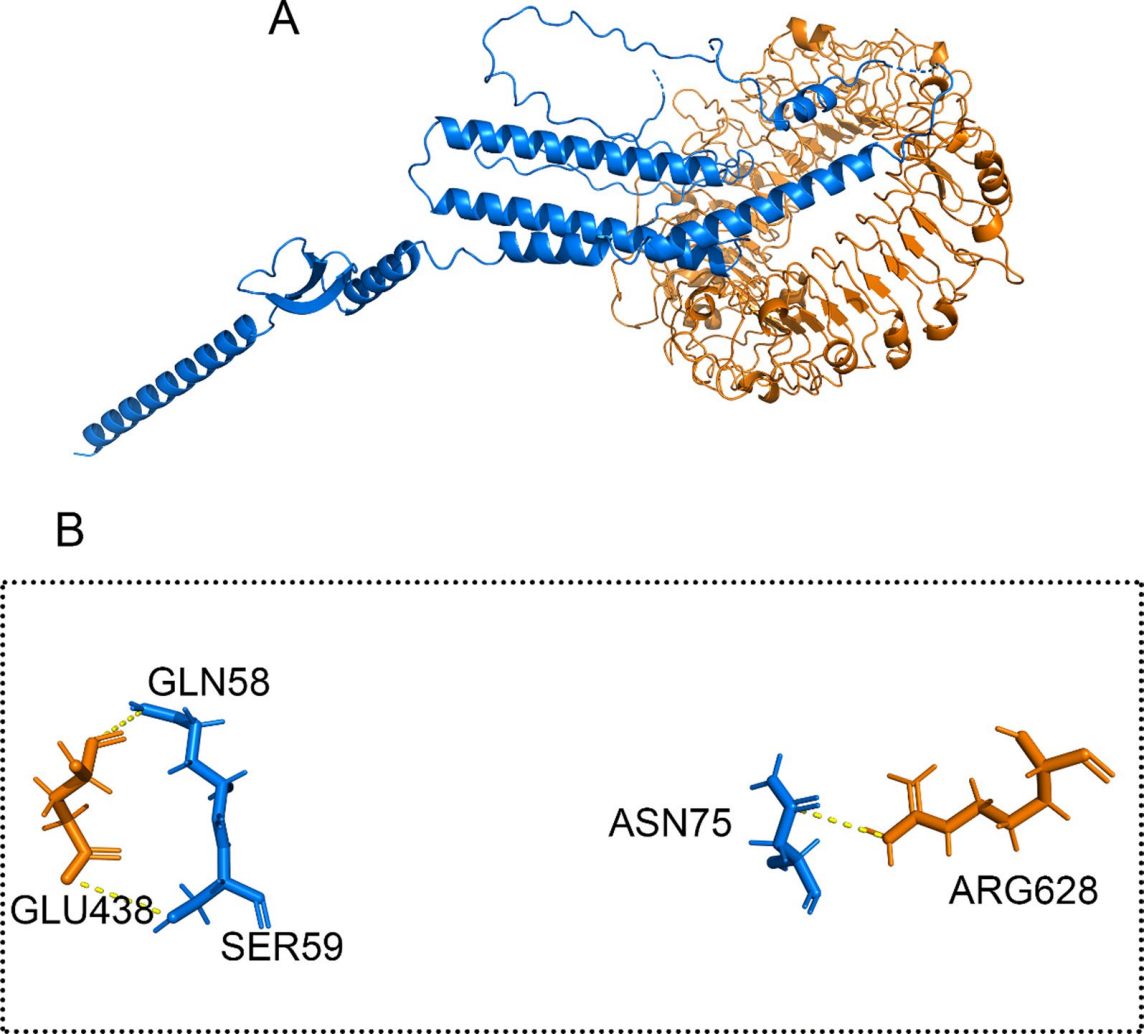
**A** Docking HADDOCK outcomes for the H5N1-TLR8 complex are visualized, **B** and the residues' contact with the complex has been magnified. An illustration of the H5N1 structure is a carton filled with green sticks. The cooper carton and sticks that make up the TLR8 structure are displayed. The dashed line in yellow represents hydrogen bonds

The docking results indicate that H5N1 is capable of forming interactions with the 3D structures of TLR7 and TLR8. Within the final construct, two sequences, PADRE and HβD-3, were selected and included as adjuvants at the C-terminal. As displayed in Figs. 6A and 7A, the initial amino acid regions of H5N1 were observed to be engaged with TLR7 and TLR8. Moreover, H5N1 directly established interactions with several amino acids within the binding sites of TLR7 and TLR8. This demonstrates the potential for a strong interaction between H5N1 and these immune-related receptors.

As illustrated in Figs. 6B and 7B, it was observed that specific amino acids in H5N1, including SER846, SER850, SER860 and PHE864 interacted with amino acids in the TLR7, such as ARG232, TYR224, PHE214 and LEU211. Also, GLN58, SER59 and ASN 75 amino acids from the H5N1 structure interacted with GLU438 and ARG628 amino acids of the TLR8 structure. This interaction demonstrates the potential binding between H5N1 and the TLR7 and TLR8.

The H5N1 exhibited interaction with TLR7 through 4 hydrogen bonds. Similarly, in the H5N1-TLR8 complex, interactions were facilitated by 3 hydrogen bonds to the interactions in this complex. These findings demonstrate the significance of hydrogen bond contacts in forming these complexes.

**Fig. 8:**
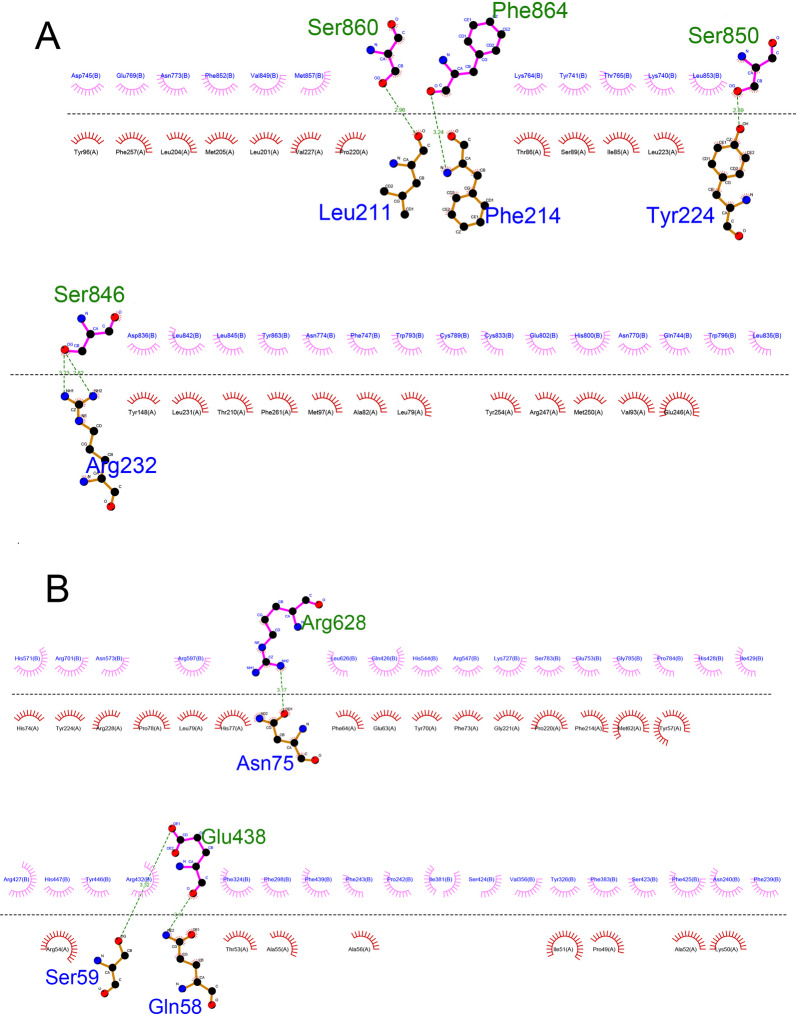
**A** Presentation of amino acids’ 2D interaction in H5N1-TLR7 complex. Green residues are presented as H5N1 structure and blue residues are presented as TLR7 structure. **B** Presentation of 2D interaction of amino acids in H5N1-TLR8 complex. Green residues are presented as H5N1 structure and blue residues are presented as TLR8 structure

The 2D interactions are summarized and visualized, providing a detailed depiction of these interactions (H5N1-TLR7 in Fig. 8A and H5N1-TLR8 in Fig. 8B).

### Molecular dynamic and MM/PBSA analysis

**Fig. 9:**
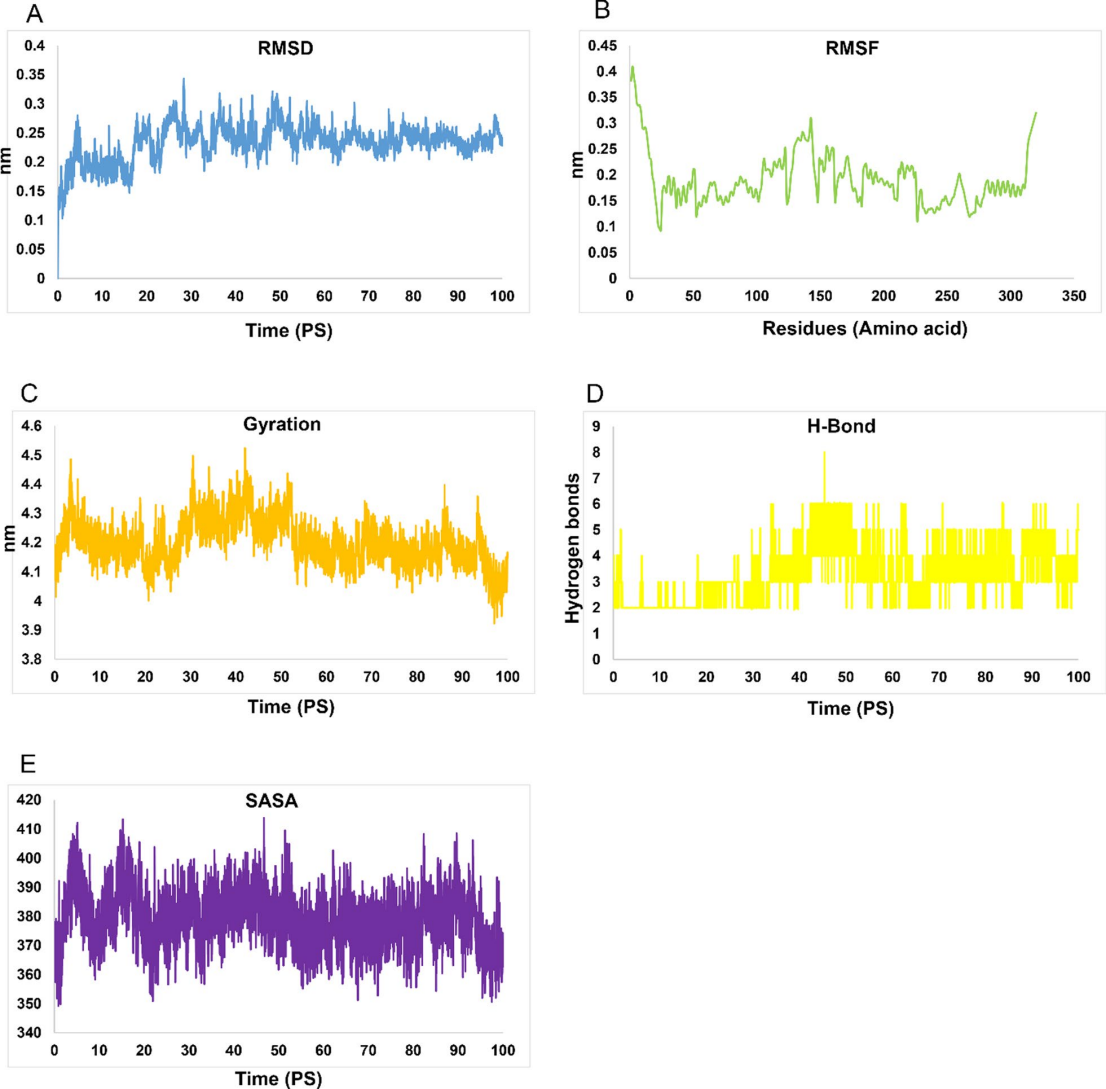
MD simulation outcomes of H5N1 complex. **A** RMSD of H5N1-TLR7 in Pico seconds, **B** RMSD of H5N1-TLR7 in residues, **C** Gyration of H5N1-TLR7 in Pico seconds, **D** H-bond of H5N1-TLR7 in Pico seconds and **E** SASA graphs of H5N1-TLR7 complex

**Fig. 10:**
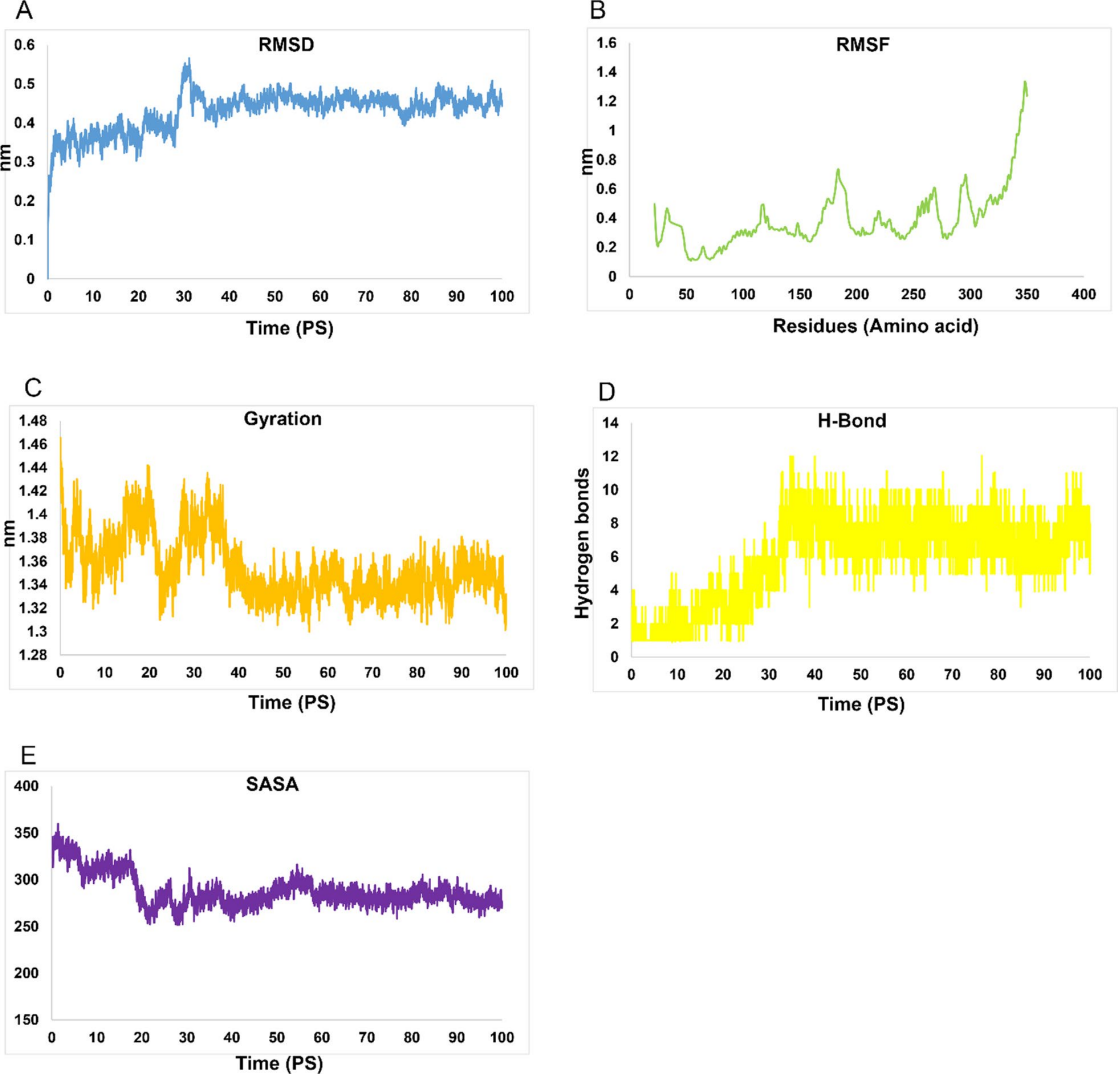
MD simulation outcomes of H5N1-TLR8 complex. **A** RMSD of H5N1-TLR8 in Pico seconds, **B** RMSD of H5N1-TLR8 in residues, **C** Gyration of H5N1-TLR8, Pico seconds (**D**) H-bond of H5N1-TLR8 in residues and **E** SASA graphs of H5N1-TLR8 complex

The complexes underwent molecular dynamics (MD) simulations for a duration of 100 ns. The MD simulation results indicated the hydrogen bonds’ creation in H5N1-TLR7 and H5N1-TLR8 complexes with root mean square deviations (RMSD) of 2 and 3.5 nm, respectively, as shown in Figs. 9A and 10A. These RMSD values provide insights into the stability and behavior of the simulated complexes during the MD simulations.

The root mean square fluctuation (RMSF) diagrams revealed that residues in the H5N1-TLR7 and H5N1-TLR8 complexes during MD simulation exhibited very weak volatility, as depicted in Figs. 9B and 10B.

These results suggest that the H5N1 structure is maintained in the binding interactions with TLR7 and TLR8 complexes. Additionally, it's worth noting that the H5N1-TLR7 complex returned to equilibrium after approximately 2 ns, while the and H5N1-TLR8 complex reached equilibrium around 3 ns after the start of the MD simulations. These findings indicate the stability and equilibration of the simulated complexes throughout the simulations.

The gyration radius data associated with H5N1-TLR7 and H5N1-TLR8 complexes can be found in Figs. 9C and 10C, providing insights into the compactness and overall structure of these complexes. Additionally, the H-bond and SASA overall results of H5N1-TLR7 and H5N1-TLR8 complexes are included in Figs. 9D and 10D as well as Figs. 9E and 10E, respectively, offering further details about the interactions within these complexes.

The H5N1-TLR7 and H5N1-TLR8 complexes' binding affinities were verified using the use of Molecular Mechanics/Poisson-Boltzmann Surface Area (MM/PBSA) modeling. Tables 10 and 11 provide an overview of the findings of these calculations, which identify the energy connected to the bonds that are created in each complex between the vaccine and the receptor. In the H5N1-TLR7 complex, the total binding energy was calculated to be − 29.97 kJ/mol, indicating a strong affinity between H5N1 and TLR7. On the other hand, in the H5N1-TLR8 complex, the total binding energy was found to be − 23.9 kJ/mol. These calculations provide insights into the thermodynamic stability and binding affinities of these complexes, with a notably high affinity observed in the case of H5N1-TLR7.

**Table 10:** MMPB/SA calculation from H5N1-TLR7 complex

Frames	VDWAALS	EEL	EPB	ENPOLAR	GGAS	GSOLV	TOTAL
Average	− 43.49	− 100.18	120.04	− 6.34	− 143.67	113.7	− 29.97
SD	3.48	15.79	16	0.57	17.15	15.75	2.5
SEM	1.05	4.76	4.83	0.17	5.17	4.75	0.75

**Table 11:** MMPB/SA calculation from H5N1-TLR8 complex

Frames	VDWAALS	EEL	EPB	ENPOLAR	GGAS	GSOLV	TOTAL
Average	− 38.06	− 132.02	151.68	− 5.49	− 170.08	146.19	− 23.9
SD	2.87	11.26	10.45	0.32	12.09	10.26	3.17
SEM	0.87	3.39	3.15	0.1	3.65	3.09	0.95

**Fig. 11:**
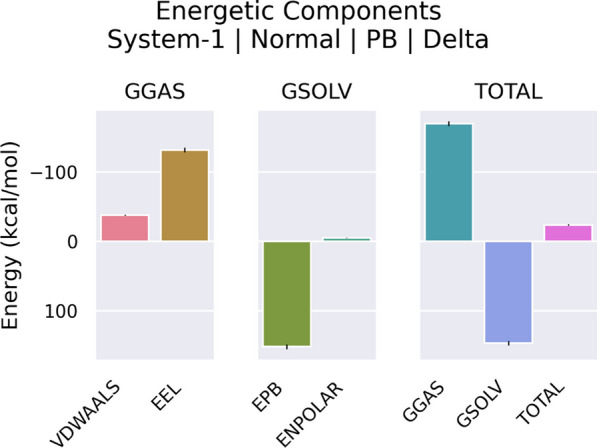
Graphical presentation of energy contribution of H5N1-TLR7 complex. Vander Waals (VDWAALS) energies are shown as pink, Electro static (EEL) energies are shown as brown, effective polarizable bonds (EPB) are as green, effective non-polarizable bonds (ENPOLAR) are shown as pale-green, gas phases (GGAS) are shown as blue, solvent phases (GSOLV) are shown as purple and total energies contribution (TOTAL) are shown as hot-pink

**Fig. 12:**
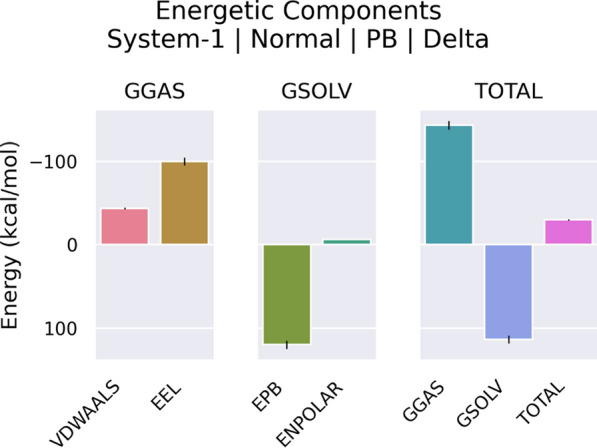
Graphical presentation of energy contribution of H5N1-TLR8 complex. Vander Waals (VDWAALS) energies are shown as pink, Electro static (EEL) energies are shown as brown, effective polarizable bonds (EPB) are as green, effective non-polarizable bonds (ENPOLAR) are shown as pale-green, gas phases (GGAS) are shown as blue, solvent phases (GSOLV) are shown as purple and total energies contribution (TOTAL) are shown as hot-pink

Additional investigations have shown that apart from total binding energy, van der Waals and electrostatic energies also have a substantial part in influencing complexes. In the H5N1-TLR7 complex, according to Table 10 and Fig. 11, the electrostatic energy has been determined to be − 100.18 kJ/mol, and the van der Waals energy to be − 43.49 kJ/mol. Conversely, in the H5N1-TLR8 complex, the van der Waals energy contribution was − 38.06 kJ/mol and the electrostatic energy was − 132.02 kJ/mol (Table 11 and Fig. 12).

These energy components further characterize the nature of the contacts in complexes, with both van der Waals and electrostatic forces playing a role in influencing the binding affinities and stability of these complexes. The balance of van der Waals as well as electrostatic energies contributes to the overall stability of these interactions.

### Immune simulation

**Fig. 13:**
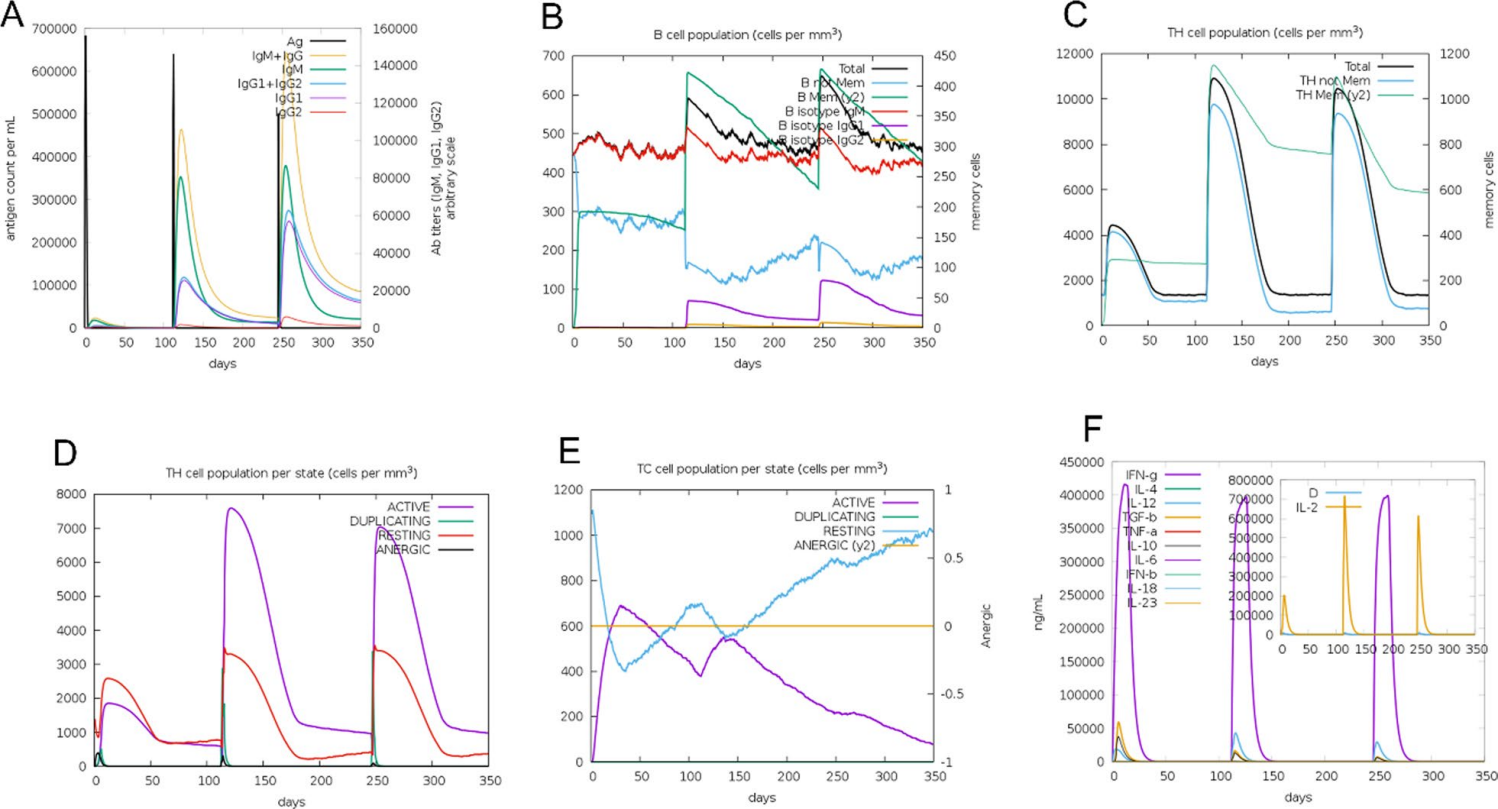
Immune response simulation against the H5N1 strain. **A** Immunoglobulin levels upon H5N1 construct. **B** Total B isotype (IgM, IgG1, IgG2) as the immunological response, and amount of B memory (y2). **C** CD4 T-helper cells as a result of the development of T-helpers. **D** Displaying both memory and total T-helper cells. **E** Cell populations of CD8 T-cytotoxic lymphocytes in each condition after being injected with antigen. **F** Generated IL-4, IL-6, and IL-12 concentrations together with IFN-γ

The C-ImmSim web server's results showed a rise in secondary immune response generation that is consistent with the true immunological response. Notably, As the immunological response grew more intense, there was a rise in total IgM + IgG1 and IgG2 antibody levels, as depicted in Fig. 13A. Moreover, the quantity of B memory (y2) increased as the levels of IgG1 and IgG2 isotypes decreased, as depicted in Fig. 13B. Additionally, the outcomes demonstrated that an increase in TH memory (y2), as depicted in Fig. 13C, correlated with an increase in both TH and TC cell populations, as illustrated in Fig. 13D. Furthermore, there was an elevation in the focus of produced IFN-γ and an increase in TH cell population, as seen in Fig. 13E, F. Together, these results show that the C-ImmSim server's simulation of the immunological response to influenza H5N1 vaccination closely resembles the real immune response, underscoring the simulation's accuracy in simulating the activity of the immune system.

### Insilico cloning and vaccine optimization

**Fig. 14:**
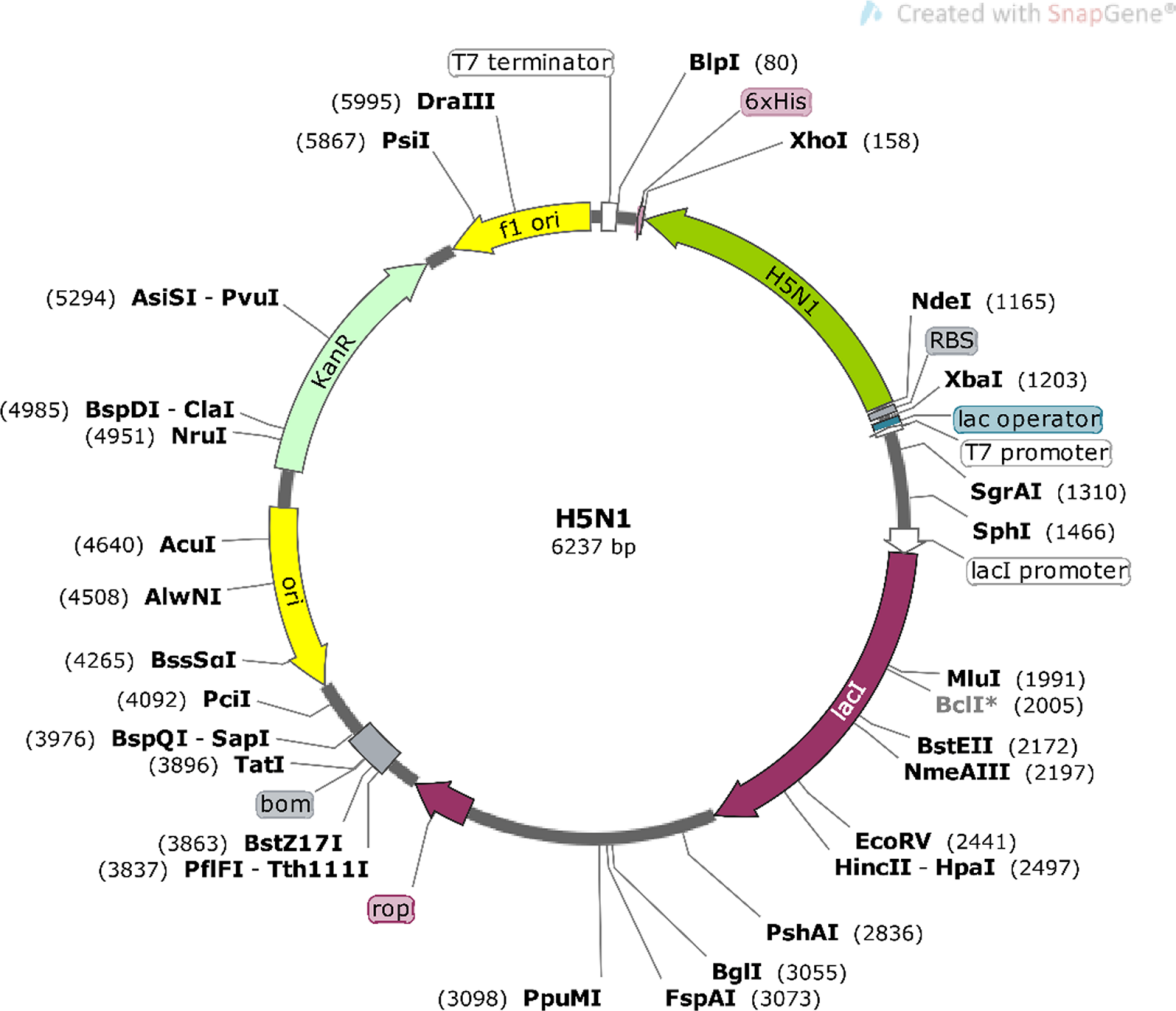
Cloned nucleotide sequence of H5N1 into pET-26b(+) plasmid. The H5N1 sequence is labeled as a green ribbon. NdeI and XhoI enzymes are located at N-terminal and C-terminal of H5N1 construct. His-Tag (6xHis) sequence is located at the C-terminal of H5N1 construct

The nucleotide sequences of the finished construct were optimized using GenScript. The organism chosen for host expression was E. Coli K12. To prevent bacterial ribosome binding sites, cleavage sites for restriction enzymes, and rho-independent transcription terminators, certain settings were made in GenScript. GC-Content for the improved Influenza construct, which consists of 1032 nucleotides (excluding the His-Tag), was determined to be 54.46%. These values affirm the likelihood of successful protein expression. To facilitate intracellular expression, to exclude the PelB sequence from the final structure, *Nde* I and *Xho* I restriction enzyme sites have been introduced to the N- and C-terminals of the nucleotide sequence, respectively. Additionally, a stop codon has been included following the His-Tag sequence. Ultimately, SnapGene was used to clone the design into the pET-26b(+) plasmid, as shown in Fig. 14.

## Discussion

Influenza H5 viruses are responsible for respiratory infections in humans, and the severity of these infections can vary from no symptoms to being life-threatening. In the realm of infectious diseases, the influenza virus stands out as among the most substantial hazards to both human and avian populations worldwide. Virologists have issued a warning over the possibility of a brand-new, extremely damaging influenza pandemic, highlighting the pressing necessity of being ready and vigilant [27]. In the two decades following its initial emergence in China in 1996, approximately 60 nations have documented ongoing cases of H5 viruses affecting domestic poultry, wild bird populations, and human beings. This underscores the widespread and continuous nature of H5 virus incidents around the world [28, 29].

Recent progress in the fields of immunoinformatics, bioinformatics, and structural vaccinomics has led to a significant transformation in how antigens are identified. These advancements have also facilitated the development of the novel vaccines creation approach, enabling the vaccines targeting a broad spectrum of bacterial and viral infections. Our prior research, has demonstrated that multi-epitope peptide vaccines can use more diverse and higher effective factors against infectious agents compared to vaccines based on single epitopes [16, 30]. In line with this approach, the present study concentrated on multiepitope subunit vaccines. These vaccines are performed using multiple immunogenic factors of pathogens and possess the capability to direct the humoral immunity reaction towards particular antigenic epitopes, ultimately leading to a safer and more efficient immune response.

In this current work, we employed the NCBI database to obtain the sequences of two viral HA/NA proteins from AIV (H5N1) strain, following a comprehensive review of the existing literature. Two viral proteins, namely HA (GenBank: BAL61222.1) and NA (GenBank: BAL61230.1), serve as surface glycoproteins responsible for binding to host cells; are two of the proteins among the most abundant within the influenza A virus and play crucial functional and structural parts throughout life cycle of viruses [31].

Vaccines stimulate B cells to generate antibodies that, in turn, carry out effector functions by specifically interacting with pathogens or toxins. Due to their role in preserving memory cells, providing longer-term immunity, and defending versus reinfection, the humoral immunity reaction plays a vital part in safeguarding versus infections through vaccination. Numerous antigens and vaccinations also cause T-cell responses in addition to B-cell responses [32–34].

Well-known CD4^+^ T cell is another crucial type of immune cell capable of adopting Th1 or Th2 phenotypes as well as orchestrating immune reactions [35]. The Th1 response is responsible for activating cytotoxic CD8^+^ T lymphocytes natural killer cells, as well as macrophages. In contrast, the Th2 response has a crucial part in activating B cells, facilitating isotype switching, promoting affinity maturation, as well as generating antibodies that can target and neutralize external pathogens [36]. T cell epitope-based vaccination is therefore a unique strategy for generating a strong immune response against pathogenic pathogens [37]. Here, to found possible CTL and HTL immunogenic epitopes inside HA and NA protein, we used topological screening, the MHC-I and MHC-II binding predictions, and the VaxiJen server. These epitopes have been selected according to their ability to link to a wide range of HLA-A and HLA-B alleles having high binding affinity.

In this study, we utilized tools to evaluate the allergenicity of chosen epitopes. During the immune stimulation procedure, allergenicity, which poses a significant challenge in vaccine development, is detected in many vaccine candidates, potentially leading to an "allergic" response. According to a criterion for predicting allergenicity, a sequence is deemed possibly allergenic if, when compared to known allergens, it shares the identity of at least six consecutive amino acids within an 80-amino acid window [38]. In addition to that, one important factor in the reverse vaccinology technique is population coverage. As per the results, all the predicted T cell epitopes from HA and NA are capable of providing coverage for populations across the majority of geographic regions worldwide, with rates exceeding 90%. Moreover, since MHC superfamilies are important in the creation of vaccines and pharmaceuticals, it has become possible to ascertain the functional link among MHC variants by MHC cluster analysis.

In our study, we used a total of 18 B cell epitopes, CTL and HTL. We used two algorithms of Linear B-cell epitope prediction methodology, including IEDB server as well as BepiPred 2.0 for predicting amino acid likely B-cell epitopes. While over the past few decades, numerous B-cell epitope prediction methods have been developed, but they have mostly failed, the majority of experimental epitope determination efforts have concentrated on finding a linear B-cell epitope prediction technique [39]. Furthermore, accurate prediction and a quicker, less expensive vaccine design process can both be aided by linear B-cell epitope prediction [40].

Based on the Bepipred linear epitope prediction 2.0 for the AIV-A (H5N1) strain, the most effective B cell epitopes for each of the three proteins were selected as possible vaccination candidates. Linear epitopes from B cells, epitopes recognized by cytotoxic T cells (CTL) and helper T cells (HTL), along with Beta-defensin 2, as well as the PADRE peptide sequence, have been employed in the formulation of the ultimate vaccine proteins. In the past, adjuvants have been used as immunomodulators to increase the effectiveness of certain vaccination candidates [41–43]. The effectiveness of the Beta-defensin adjuvant as an immune booster has been demonstrated in numerous experiments against various organisms [44–46]. The VaxiJen server has been utilized to evaluate all of the acquired protein sequences, determining the most potential antigenic protein with the capacity to induce immunity.

AAY linkers have been utilized to bond CTL (Cytotoxic T Lymphocyte) epitopes to each other. These linkers help separate and align CTL epitopes effectively. Specific HEYGAEALERAG linker serves as the interface among CTL epitopes as well as HTL (Helper T Lymphocyte) epitopes, ensuring a proper connection and interaction between these two critical components of the vaccine. GPGPG linkers act as connectors between different HTL epitopes. They help maintain structural integrity and spacing within the HTL epitope region. KK linkers have been used to connect B-cell epitopes, as well as have been utilized to link HβD-3 and PADRE adjuvants in the N-terminal of the construct. These linkers ensure that B-cell epitopes are properly connected, enhancing their accessibility for immune recognition. EAAAK was used to bond the vaccine construct to the HisTag sequence at the C-terminal. EAAAK Linkers help maintain flexibility and proper spacing between these components. The strategic use of these linkers is essential in vaccine design to ensure that different epitopes and components work together efficiently, promoting a robust immune response when administered.

Additionally, The ProtParam server has been utilized to examine the final construct's physicochemical characteristics. The molecular weight of the H5N1 vaccine construct is 38.46 kDa. This is deemed a suitable vaccine candidate, as proteins having a molecular weight of less than 110 kDa are generally regarded as more favorable for vaccine development because of their ease of purification [47]. After expression, the construct's heat stability was evaluated using the aliphatic index. The H5N1 protein is classified as stable by the estimated instability index since its value is less than 40. An instability index of less than 40 indicates stability, whereas a value above 40 suggests potential instability. The calculated GRAVY index of H5N1 serves as an indicator of its polar nature and its interaction with water. A greater solubility for this structure is indicated by a lower GRAVY score. Furthermore, the selected vaccine candidate exhibited a higher proportion of coil structure in its secondary structure. This suggests that random coils are significant in conferring greater protein flexibility and might contribute to an enhanced antibody binding capability [48].

Incorporating adjuvants into the subunit vaccine could enhance the immunity reaction it elicits. Adjuvants serve to be activators for Toll-like receptors (TLRs) and could be effectors to broaden antibody recognition [49]. It was observed that TLR7 and TLR8 are capable of recognizing Influenza Vaccine Antigen (IVA) due to their ability to couple with single-stranded RNAs (ssRNAs) [50]. TLR-7 was demonstrated to trigger both a humoral and long-lasting memory response in reaction to ssRNA-mediated infections as well as vaccinations, such as those involving Influenza and HIV [51, 52]. Additionally, It has been discovered that synthetic TLR7 and TLR8 agonists enhance both innate and adaptive immune responses [53].

The interaction between H5N1 3D structure and TLR7 and TLR8 structures has been examined through protein–protein docking. In the case of H5N1-TLR7 as well as H5N1-TLR8 complexes, it was observed that these interactions involved 4 and 6 hydrogen bonds, respectively.

After docking and determining how to connect complexes H5N1-TLR7 and H5N1-TLR8 we performed the MD test. The MD technique was performed to check the stability and behavior of the complexes per unit of time. Since it is possible that over time, the proteins in a complex move away from each other and the complex becomes unstable, MD can show the binding quality of two proteins resulting from their docking (within a certain time).

So in our study to do MD:

1. First, we ran each complex separately for 100 ps and analyzed the results through RMSD, RMSF, Gyration, H-bond and SASA graphs.
2. Then we went one step further and analyzed the MD results using MMPBSA to estimate the contribution of each of the energies involved in the complex such as electrostatics, van der Waals, etc.

The complexes of H5N1 with TLR7 and TLR8 demonstrated interactions between the proteins via the creation of hydrogen bonds. Analysis of RMSD diagrams for the H5N1-TLR7 and H5N1-TLR8 complexes revealed that the simulations were relatively stable. However, it's noteworthy that Compared to the H5N-TLR8 complex, which had a mean RMSD of 0.3 nm, the H5N1-TLR7 complex had a mean RMSD of 0.6 nm, indicating that its intermolecular energies are higher. The stability of the complex is confirmed by the gyration graph, which shows that the amino acids stay inside and don't go beyond their radial axis range during the simulation. Furthermore, the RMSF plot aligns with these outcomes, indicating that the amino acids within binding sites of H5N1-TLR7 and H5N1-TLR8 complexes keep their stability during simulation.

Overall, the results from the docking and molecular dynamics (MD) simulations suggest that these complexes are capable of maintaining their stability during the simulation. The outcomes from such a study indicate that recombinant construct may effectively stimulate TLR7 and TLR8. The fact that TLR7 and TLR8 attach to the residues in the vaccine structure suggests that the vaccination suggested in this study would probably cause the development of antibodies against it. This expectation stems from the importance of having the accurate 3D structure of antigen for it to be identified effectively via the humoral immunity mechanism. Consequently, all selected epitopes within the 3D-vaccine structure should closely mimic the correct fold found in the native structure of the antigen to elicit a comparable immunity response [54].

Additionally, the MM/PBSA outcomes revealed that, apart from the overall energy, the complexes are influenced by the energies arising from electrostatic and covalent bonds. Notably, the positive binding energy observed in the H5N1-TLR8 complex could be influenced by presence of salt bridges in contact between the TLR8 and vaccine structure [55].

Influenza viruses are ssRNA viruses that require the activation of T cell-dependent immune responses plus humoral immunity induced via B cells. This activation is crucial for preventing the proliferation and intracellular survival of the virus. According to predictions from the C-ImmSim Online server, the growth of both B cells and T cells leads to the establishment of long-term immune memory. Thus, IgG2 and IgG1 which are indicative of Th2 and Th1 responses to influenza antigens, respectively, play important roles in protecting against influenza infection [56, 57]. Past research has indicated that both avian and human hosts experience a reduction in their T-cell populations in reaction to avian influenza infection [58]. A decrease in T lymphocytes is frequently linked to proinflammatory cytokines’ upregulation, particularly interferons (IFNs), notably IFN-γ, and interleukins (ILs) like IL-6. These changes can, in the end, result in a condition called hypercytokinemia and trigger the cell apoptosis’ activation [59]. Increased levels of IL-12 and IFN-γ are indicative of a robust cellular immune response. While previous research has demonstrated that T epitopes from H5N1 strains designed to mimic human sequences may lead to weakened IFN responses in humans, the results from the ICM server indicate that this vaccine has an excellent capacity to stimulate IFN-γ, suggesting its potential to trigger a robust immune response [60–64]]. Nonetheless, the findings from the ICM server suggest that a rise in the count of Th1 cells is linked to the elevation in the number of cytotoxic T cells.

### Supplementary Information

**Additional file 1:** Supplementary.

## Data Availability

Not applicable.
