# Supplementary material for: A computational approach to design a multiepitope vaccine against H5N1 virus
Source: Virol J. 2024 Mar 20;21:67. doi: 10.1186/s12985-024-02337-7 (PMC10953225; doi:10.1186/s12985-024-02337-7)
Supplement: Supplementary file 1 — Additional file 1: Supplementary. [file 12985_2024_2337_MOESM1_ESM.docx]

**Table S1.**B cell epitopes predicted from HA protein.

| **No.** | **Start** | **End** | **Peptide** | **Vaxigen** |
| --- | --- | --- | --- | --- |
| 1 | 29 | 32 | TEQV | - |
| 2 | 40 | 70 | VTVTHAQDILEKTHNGKLCDLDGVKPLILRD | 0.3738 |
| 3 | 82 | 90 | MCDEFINVP | -0.1833 |
| 4 | 100 | 156 | NPNNDLCYPGSFNDYEELKHLLSRINHFEKIQIIPKNSWSDHEASSGVSAACPYLGS | 0.4128 |
| 5 | 169 | 169 | K | - |
| 6 | 171 | 185 | STYPTIKKSYNNTNQ | 0.2372 |
| 7 | 200 | 211 | AAEQTRLYQNPT | 0.2454 |
| 8 | 223 | 240 | QRLVPKIATRSKVNGQSG | 0.7994 |
| 9 | 274 | 296 | KKGDSAIMKSELEYGNCNTKCQT | 0.7166 |
| 10 | 299 | 310 | GAINSSMPFHNI | 0.4388 |
| 11 | 330 | 346 | ATGLRNSPQRESRRKKR | 0.4021 |
| 12 | 359 | 397 | GWQGMVDGWYGYHHSNEQGSGYAADKESTQKAVDGVTNK | 0.2432 |
| 13 | 399 | 399 | N | - |
| 14 | 402 | 402 | I | - |
| 15 | 407 | 407 | T | - |
| 16 | 410 | 420 | EAVGREFNNLE | 0.7565 |
| 17 | 430 | 432 | MED | - |
| 18 | 452 | 460 | RTLDFHDSN | 1.7355 |
| 19 | 463 | 463 | N | - |
| 20 | 476 | 481 | AKELGN | 0.2864 |
| 21 | 495 | 527 | MESIRNGTYNYPQYSEEARLKREEINGVKLESV | 0.5040 |

**Table S2.** B cell epitopes predicted from HA protein.

| **No.** | **Start** | **End** | **Peptide** | **Vaxigen** |
| --- | --- | --- | --- | --- |
| 1 | 38 | 55 | IQKGNQHQAESISNTNPL | 0.7037 |
| 2 | 68 | 74 | NSSLCPI | 1.3010 |
| 3 | 119 | 134 | LMNDKHSNGTVKDRSP | 0.8291 |
| 4 | 144 | 147 | GEAP | - |
| 5 | 193 | 206 | TDTIKSWRNNILRT | -0.9533 |
| 6 | 226 | 236 | PSNGQASYKIF | 0.3125 |
| 7 | 240 | 252 | KGKVVKSVELDAP | 0.0972 |
| 8 | 276 | 276 | W | - |
| 9 | 303 | 323 | GDNPRPNDGTGSCGPMSPNGA | 0.1778 |
| 10 | 334 | 335 | GN | - |
| 11 | 337 | 337 | V | - |
| 12 | 342 | 370 | TKSTNSRSGFEMIWDPNGWTGTDSSFSVK | 0.7274 |
| 13 | 376 | 381 | ITDWSG | -1.0444 |
| 14 | 391 | 392 | EL | - |
| 15 | 394 | 394 | G | - |
| 16 | 415 | 415 | T | - |
| 17 | 431 | 445 | DTVSWSWPDGAELPF | 0.5551 |

**Table S3.** CTL cell epitopes predicted from HA protein

| HLA | peptide | 1-log50k(aff) | Affinity(nM) | %Rank | Bind | Level | Vaxijen |
| --- | --- | --- | --- | --- | --- | --- | --- |
| HLA-A0101 | KSDQICIGY | 0.664 | 38.08 | 0.07 | <= | SB | **2.07** |
| HLA-A0201 | FLDVWTYNA | 0.852 | 4.96 | 0.03 | <= | SB | **-0.7897** |
| HLA-A0202 | MMAGLSLWM | 0.852 | 4.95 | 0.06 | <= | SB | **0.1822** |
| HLA-A0203 | LLLAMVSLV | 0.822 | 6.88 | 0.17 | <= | SB | **0.8565** |
| HLA-A0205 | IAPEYAYKI | 0.343 | 1225.26 | 0.3 | <= | SB | **0.9842** |
| HLA-A0206 | KMEDGFLDV | 0.753 | 14.47 | 0.3 | <= | SB | **0.8246** |
| HLA-A0207 | DLDGVKPLI | 0.219 | 4664.55 | 0.09 | <= | SB | **-0.4645** |
| HLA-A0211 | LLLAMVSLV | 0.887 | 3.39 | 0.1 | <= | SB | **0.8565** |
| HLA-A0212 | FLDVWTYNA | 0.903 | 2.85 | 0.01 | <= | SB | **-0.7897** |
| HLA-A0216 | FLDVWTYNA | 0.909 | 2.68 | 0.01 | <= | SB | **-0.7897** |
| HLA-A0217 | SLAIMMAGL | 0.565 | 110.58 | 0.2 | <= | SB | **0.6432** |
| HLA-A0219 | LLLAMVSLV | 0.864 | 4.37 | 0.03 | <= | SB | **0.8565** |
| HLA-A0250 | KMEDGFLDV | 0.879 | 3.7 | 0.02 | <= | SB | **0.8246** |
| HLA-A0301 | RLYQNPTTY | 0.67 | 35.5 | 0.17 | <= | SB | **-0.2918** |
| HLA-A1101 | FIAPEYAYK | 0.708 | 23.62 | 0.15 | <= | SB | **0.8471** |
| HLA-A2301 | PYLGSPSFF | 0.718 | 21.19 | 0.07 | <= | SB | **0.2991** |
| HLA-A2402 | PYLGSPSFF | 0.62 | 60.92 | 0.12 | <= | SB | **0.2991** |
| HLA-A2403 | CYPGSFNDY | 0.87 | 4.07 | 0.04 | <= | SB | **0.5962** |
| HLA-A2501 | GTYQILSIY | 0.339 | 1270.34 | 0.15 | <= | SB | **0.466** |
| HLA-A2601 | DAAEQTRLY | 0.666 | 37.19 | 0.06 | <= | SB | **0.1686** |
| HLA-A2602 | DAAEQTRLY | 0.73 | 18.62 | 0.09 | <= | SB | **0.1686** |
| HLA-A2603 | HSNEQGSGY | 0.448 | 393.65 | 0.06 | <= | SB | **0.1510** |
| HLA-A2902 | GMVDGWYGY | 0.888 | 3.37 | 0.01 | <= | SB | **0.217** |
| HLA-A3001 | LSRINHFEK | 0.748 | 15.27 | 0.12 | <= | SB | **0.173** |
| HLA-A3002 | GTYQILSIY | 0.686 | 30 | 0.07 | <= | SB | **0.466** |
| HLA-A3101 | RLVPKIATR | 0.794 | 9.27 | 0.08 | <= | SB | **1.009** |
| HLA-A3201 | RMEFFWTIL | 0.816 | 7.29 | 0.01 | <= | SB | **1.195** |
| HLA-A3207 | MPFHNIHPL | 0.735 | 17.58 | 0.03 | <= | SB | **1.263** |
| HLA-A3215 | MPFHNIHPL | 0.696 | 26.87 | 0.01 | <= | SB | **1.263** |
| HLA-A3301 | YLGSPSFFR | 0.7 | 25.67 | 0.09 | <= | SB | **-0.232** |
| HLA-A6601 | MPFHNIHPL | 0.363 | 986.12 | 0.01 | <= | SB | **1.2633** |
| HLA-A6801 | FIAPEYAYK | 0.79 | 9.69 | 0.09 | <= | SB | **0.8471** |
| HLA-A6802 | NVPEWSYIV | 0.723 | 20.06 | 0.3 | <= | SB | **0.0733** |
| HLA-A6823 | MPFHNIHPL | 0.79 | 9.68 | 0.01 | <= | SB | **1.2633** |
| HLA-A6901 | NVPEWSYIV | 0.753 | 14.48 | 0.08 | <= | SB | **0.0733** |
| HLA-A8001 | AIMKSELEY | 0.689 | 28.91 | 0.03 | <= | SB | **0.3738** |
| HLA-B0702 | SPSFFRNVV | 0.71 | 23.14 | 0.12 | <= | SB | **-0.4537** |
| HLA-B0801 | MPFHNIHPL | 0.659 | 40.2 | 0.12 | <= | SB | **1.2633** |
| HLA-B0802 | HLLSRINHF | 0.25 | 3345.29 | 0.03 | <= | SB | **0.2907** |
| HLA-B0803 | YVKSNRLVL | 0.231 | 4094.51 | 0.07 | <= | SB | **0.1182** |
| HLA-B1402 | MPFHNIHPL | 0.363 | 980.92 | 0.17 | <= | SB | **1.2633** |
| HLA-B1501 | GAINSSMPF | 0.744 | 15.97 | 0.12 | <= | SB | **0.4755** |
| HLA-B1502 | GMVDGWYGY | 0.641 | 48.76 | 0.02 | <= | SB | **0.2167** |
| HLA-B1503 | RLYQNPTTY | 0.817 | 7.27 | 0.08 | <= | SB | **-0.2918** |
| HLA-B1509 | FHDSNVKNL | 0.65 | 43.95 | 0.01 | <= | SB | **0.6013** |
| HLA-B1517 | STVASSLSL | 0.895 | 3.11 | 0.06 | <= | SB | **0.7044** |
| HLA-B1801 | DEFINVPEW | 0.844 | 5.43 | 0.01 | <= | SB | **-0.5278** |
| HLA-B2705 | GRMEFFWTI | 0.74 | 16.64 | 0.05 | <= | SB | **1.2086** |
| HLA-B2720 | GRMEFFWTI | 0.8 | 8.67 | 0.04 | <= | SB | **1.2086** |
| HLA-B3501 | MPFHNIHPL | 0.834 | 6.01 | 0.03 | <= | SB | **1.2633** |
| HLA-B3503 | CPYLGSPSF | 0.321 | 1547.44 | 0.05 | <= | SB | **1.1181** |
| HLA-B3801 | FHNIHPLTI | 0.453 | 370.07 | 0.12 | <= | SB | **1.3066** |
| HLA-B3901 | MPFHNIHPL | 0.825 | 6.61 | 0.01 | <= | SB | **1.2633** |
| HLA-B4001 | REEINGVKL | 0.84 | 5.66 | 0.01 | <= | SB | **0.4733** |
| HLA-B4002 | FESNGNFIA | 0.639 | 49.64 | 0.15 | <= | SB | **0.1115** |
| HLA-B4013 | RMEFFWTIL | 0.543 | 140.81 | 0.05 | <= | SB | **1.1954** |
| HLA-B4201 | MPFHNIHPL | 0.743 | 16.2 | 0.01 | <= | SB | **1.2633** |
| HLA-B4402 | MENERTLDF | 0.764 | 12.92 | 0.01 | <= | SB | **1.5311** |
| HLA-B4403 | MENERTLDF | 0.68 | 32.05 | 0.04 | <= | SB | **1.5311** |
| HLA-B4501 | NEQGSGYAA | 0.68 | 31.96 | 0.04 | <= | SB | **0.2938** |
| HLA-B4601 | GAINSSMPF | 0.483 | 268.14 | 0.03 | <= | SB | **0.4755** |
| HLA-B4801 | RMEFFWTIL | 0.492 | 244.69 | 0.02 | <= | SB | **1.1954** |
| HLA-B5101 | CPYLGSPSF | 0.44 | 429.32 | 0.08 | <= | SB | **1.1181** |
| HLA-B5301 | CPYLGSPSF | 0.711 | 22.92 | 0.05 | <= | SB | **1.1181** |
| HLA-B5401 | MPFHNIHPL | 0.741 | 16.45 | 0.07 | <= | SB | **1.2633** |
| HLA-B5701 | IMMAGLSLW | 0.663 | 38.34 | 0.12 | <= | SB | **0.2362** |
| HLA-B5801 | QSGRMEFFW | 0.873 | 3.97 | 0.01 | <= | SB | **1.0528** |
| HLA-B5802 | QSGRMEFFW | 0.154 | 9413.88 | 0.15 | <= | SB | **1.0528** |
| HLA-B7301 | NEQGSGYAA | 0.276 | 2518.72 | 0.08 | <= | SB | **0.2938** |
| HLA-B8301 | MPFHNIHPL | 0.576 | 97.78 | 0.01 | <= | SB | **1.2633** |
| HLA-C0303 | YAADKESTQ | 0.803 | 8.44 | 0.07 | <= | SB | **0.3498** |
| HLA-C0401 | MEFFWTILK | 0.275 | 2547.47 | 0.07 | <= | SB | **0.5463** |
| HLA-C0501 | IIDKMNTQF | 0.721 | 20.49 | 0.04 | <= | SB | **0.6239** |
| HLA-C0602 | YVKSNRLVL | 0.473 | 298.4 | 0.17 | <= | SB | **0.1182** |
| HLA-C0701 | YVKSNRLVL | 0.465 | 326.16 | 0.15 | <= | SB | **0.1182** |
| HLA-C0702 | YVKSNRLVL | 0.435 | 451.64 | 0.15 | <= | SB | **0.1182** |
| HLA-C0802 | FHDSNVKNL | 0.425 | 502.87 | 0.03 | <= | SB | **0.6013** |
| HLA-C1203 | YVKSNRLVL | 0.731 | 18.45 | 0.05 | <= | SB | **0.1182** |
| HLA-C1402 | TYPTIKKSY | 0.771 | 11.94 | 0.03 | <= | SB | **0.1472** |
| HLA-C1502 | WTYNAELLV | 0.67 | 35.55 | 0.01 | <= | SB | **0.3184** |

**Table S4.** CTL cell epitopes predicted from NA protein.

| HLA | peptide | 1-log50k(aff) | Affinity(nM) | %Rank | Bind | Level | Vaxijen |
| --- | --- | --- | --- | --- | --- | --- | --- |
| HLA-A0101 | VSFNQNLEY | 0.673 | 34.28 | 0.06 | <= | SB | **0.9616** |
| HLA-A0201 | GMVSLMLQI | 0.627 | 56.48 | 0.7 | <= | WB | **0.8929** |
| HLA-A0202 | HLECRIFFL | 0.702 | 25.01 | 0.7 | <= | WB | **1.1819** |
| HLA-A0203 | LMSCPVGEA | 0.753 | 14.46 | 0.4 | <= | SB | **-0.0527** |
| HLA-A0205 | LTEKAVASV | 0.464 | 331.44 | 0.07 | <= | SB | **0.7283** |
| HLA-A0206 | LQIGNIISI | 0.82 | 7.02 | 0.09 | <= | SB | **1.2621** |
| HLA-A0207 | GLDCIRPCF | 0.118 | 13931.81 | 0.8 | <= | WB | **1.4833** |
| HLA-A0211 | ITIGSICMV | 0.895 | 3.12 | 0.08 | <= | SB | **2.0061** |
| HLA-A0212 | LQIGNIISI | 0.779 | 10.88 | 0.15 | <= | SB | **1.2621** |
| HLA-A0216 | CVNGSCFTV | 0.721 | 20.48 | 0.25 | <= | SB | **0.1636** |
| HLA-A0217 | HLECRIFFL | 0.606 | 71.01 | 0.12 | <= | SB | **1.1819** |
| HLA-A0219 | CVNGSCFTV | 0.677 | 32.77 | 0.2 | <= | SB | **0.1636** |
| HLA-A0250 | TLAGNSSLC | 0.739 | 16.88 | 0.3 | <= | SB | **0.2577** |
| HLA-A0301 | KIFKMRKGK | 0.722 | 20.27 | 0.08 | <= | SB | **-0.9793** |
| HLA-A1101 | GQASYKIFK | 0.707 | 23.84 | 0.15 | <= | SB | **0.1961** |
| HLA-A2301 | AYGVKGFSF | 0.575 | 99.25 | 0.4 | <= | SB | **1.2975** |
| HLA-A2402 | AYGVKGFSF | 0.621 | 60.32 | 0.12 | <= | SB | **1.2975** |
| HLA-A2601 | MVIGMVSLM | 0.847 | 5.23 | 0.01 | <= | SB | **1.0720** |
| HLA-A2602 | MVIGMVSLM | 0.822 | 6.85 | 0.03 | <= | SB | **1.0720** |
| HLA-A2603 | VAITDWSGY | 0.422 | 519.79 | 0.08 | <= | SB | **0.3178** |
| HLA-A2902 | GVKGFSFKY | 0.711 | 22.73 | 0.17 | <= | SB | **1.1111** |
| HLA-A3001 | KMRKGKVVK | 0.858 | 4.65 | 0.02 | <= | SB | **-0.5711** |
| HLA-A3002 | GVKGFSFKY | 0.611 | 67.34 | 0.25 | <= | SB | **1.1111** |
| HLA-A3101 | KSWRNNILR | 0.829 | 6.34 | 0.04 | <= | SB | **-1.8112** |
| HLA-A3201 | GSNRPWVSF | 0.721 | 20.52 | 0.05 | <= | SB | **0.0621** |
| HLA-A3207 | RGRPKESTI | 0.593 | 81.83 | 0.2 | <= | SB | **0.7877** |
| HLA-A3215 | DVFVIREPF | 0.483 | 268.36 | 0.07 | <= | SB | **-0.2339** |
| HLA-A3301 | YGNGVWIGR | 0.601 | 75.11 | 0.3 | <= | SB | **0.9891** |
| HLA-A6601 | MVIGMVSLM | 0.285 | 2301.39 | 0.08 | <= | SB | **1.0720** |
| HLA-A6801 | EAPSPYNSR | 0.652 | 43.35 | 0.6 | <= | WB | **0.5467** |
| HLA-A6802 | ESISNTNPL | 0.846 | 5.3 | 0.05 | <= | SB | **0.7866** |
| HLA-A6823 | YHYEECSCY | 0.633 | 53.08 | 0.12 | <= | SB | **0.5920** |
| HLA-A6901 | ESISNTNPL | 0.729 | 18.85 | 0.09 | <= | SB | **0.7866** |
| HLA-A8001 | VSFNQNLEY | 0.725 | 19.58 | 0.02 | <= | SB | **0.9616** |
| HLA-B0702 | RPNDGTGSC | 0.623 | 59.39 | 0.3 | <= | SB | **0.1459** |
| HLA-B0801 | FKMRKGKVV | 0.601 | 75.31 | 0.25 | <= | SB | **0.5004** |
| HLA-B0802 | HLECRIFFL | 0.299 | 1975.27 | 0.01 | <= | SB | **1.1819** |
| HLA-B0803 | HLECRIFFL | 0.211 | 5079.26 | 0.12 | <= | SB | **1.1819** |
| HLA-B1402 | DRSPHRTLM | 0.33 | 1410.27 | 0.3 | <= | SB | **0.0722** |
| HLA-B1501 | YQIGYICSG | 0.612 | 66.4 | 0.6 | <= | WB | **1.3358** |
| HLA-B1502 | YHYEECSCY | 0.592 | 82.52 | 0.04 | <= | SB | **0.5920** |
| HLA-B1503 | VSFNQNLEY | 0.775 | 11.44 | 0.17 | <= | SB | **0.9616** |
| HLA-B1509 | EKAVASVTL | 0.402 | 648.89 | 0.1 | <= | SB | **0.6042** |
| HLA-B1517 | VSFNQNLEY | 0.904 | 2.81 | 0.05 | <= | SB | **0.9616** |
| HLA-B1801 | NEAVAVLKY | 0.812 | 7.61 | 0.03 | <= | SB | **0.4404** |
| HLA-B2705 | IRGWAVHSK | 0.543 | 140.39 | 0.6 | <= | WB | **0.2051** |
| HLA-B2720 | HRTLMSCPV | 0.811 | 7.76 | 0.03 | <= | SB | **0.3564** |
| HLA-B3501 | WPDGAELPF | 0.857 | 4.71 | 0.01 | <= | SB | **0.0569** |
| HLA-B3503 | EPFISCSHL | 0.391 | 725.89 | 0.03 | <= | SB | **0.5449** |
| HLA-B3801 | SHLECRIFF | 0.562 | 114.17 | 0.03 | <= | SB | **0.8715** |
| HLA-B3901 | EKAVASVTL | 0.744 | 15.87 | 0.04 | <= | SB | **0.6042** |
| HLA-B4001 | LEYQIGYIC | 0.532 | 158.53 | 0.5 | <= | SB | **1.9916** |
| HLA-B4002 | LEYQIGYIC | 0.656 | 41.21 | 0.1 | <= | SB | **1.9916** |
| HLA-B4013 | FKYGNGVWI | 0.464 | 328.89 | 0.12 | <= | SB | **0.5276** |
| HLA-B4201 | EPFISCSHL | 0.654 | 42.03 | 0.04 | <= | SB | **0.5449** |
| HLA-B4402 | NEAVAVLKY | 0.684 | 30.67 | 0.06 | <= | SB | **0.4404** |
| HLA-B4403 | NEAVAVLKY | 0.688 | 29.28 | 0.03 | <= | SB | **0.4404** |
| HLA-B4501 | EECSCYPDA | 0.663 | 38.36 | 0.05 | <= | SB | **0.1255** |
| HLA-B4601 | VSFNQNLEY | 0.364 | 973.45 | 0.09 | <= | SB | **0.9616** |
| HLA-B4801 | CMVIGMVSL | 0.361 | 1001.31 | 0.1 | <= | SB | **1.9406** |
| HLA-B5101 | EPFISCSHL | 0.364 | 970.93 | 0.2 | <= | SB | **0.5449** |
| HLA-B5301 | WPDGAELPF | 0.656 | 41.13 | 0.09 | <= | SB | **0.0569** |
| HLA-B5401 | NPLTEKAVA | 0.405 | 623.14 | 0.8 | <= | WB | **0.0810** |
| HLA-B5701 | SSLCPIRGW | 0.68 | 31.87 | 0.1 | <= | SB | **0.2320** |
| HLA-B5801 | NSDTVSWSW | 0.867 | 4.21 | 0.01 | <= | SB | **0.8772** |
| HLA-B5802 | KSTNSRSGF | 0.142 | 10720.56 | 0.25 | <= | SB | **0.5758** |
| HLA-B7301 | FKYGNGVWI | 0.326 | 1466.76 | 0.04 | <= | SB | **0.5276** |
| HLA-B8301 | APSPYNSRF | 0.477 | 285.82 | 0.01 | <= | SB | **0.7486** |
| HLA-C0303 | WSWPDGAEL | 0.827 | 6.47 | 0.05 | <= | SB | **0.4257** |
| HLA-C0401 | FFLTQGALM | 0.295 | 2051.38 | 0.04 | <= | SB | **0.6121** |
| HLA-C0501 | ACHDGTSWL | 0.479 | 279.62 | 0.25 | <= | SB | **-0.5380** |
| HLA-C0602 | DRSPHRTLM | 0.596 | 78.83 | 0.05 | <= | SB | **0.0722** |
| HLA-C0701 | DRSPHRTLM | 0.624 | 58.21 | 0.02 | <= | SB | **0.0722** |
| HLA-C0702 | IRPCFWVEL | 0.402 | 644.03 | 0.25 | <= | SB | **1.1888** |
| HLA-C0802 | FSFKYGNGV | 0.372 | 891.48 | 0.06 | <= | SB | **1.3196** |
| HLA-C1203 | FSFKYGNGV | 0.77 | 12.1 | 0.03 | <= | SB | **1.3196** |
| HLA-C1402 | YHYEECSCY | 0.753 | 14.52 | 0.05 | <= | SB | **0.5920** |
| HLA-C1502 | ISIWVSHPI | 0.714 | 22.19 | 0.01 | <= | SB | **-0.4044** |

**Table S5.** HTL cell epitopes predicted from HA protein

| Allele | peptide | 1-log50k(aff) | affinity(nM) | %Rank | Relia | Bind | Vaxijen |
| --- | --- | --- | --- | --- | --- | --- | --- |
| DRB1_0101 | EFFWTILKPNDAINF | 0.8401 | 5.6 | 0.8 | 0.83 | SB | **0.5642** |
| DRB1_0103 | NLYDKVRLQLRDNAK | 0.2671 | 2778.9 | 2.5 | 0.5 | WB | **1.1390** |
| DRB1_0301 | ELLVLMENERTLDFH | 0.5868 | 87.4 | 4 | 0.44 | WB | **1.0452** |
| DRB1_0401 | PTTYISIGTSTLNQR | 0.7244 | 19.7 | 0.5 | 0.71 | SB | **1.0883** |
| DRB1_0402 | RGLFGAIAGFIEGGW | 0.4545 | 365.8 | 0.8 | 0.24 | SB | **0.1185** |
| DRB1_0403 | SSLSLAIMMAGLSLW | 0.418 | 542.7 | 0.7 | 0.34 | SB | **0.5652** |
| DRB1_0404 | AGLSLWMCSNGSLQC | 0.6891 | 28.9 | 1.5 | 0.67 | SB | **0.2611** |
| DRB1_0405 | AELLVLMENERTLDF | 0.7215 | 20.4 | 0.4 | 0.83 | SB | **1.0504** |
| DRB1_0701 | IYSTVASSLSLAIMM | 0.83 | 6.3 | 0.4 | 0.81 | SB | **0.5702** |
| DRB1_0801 | ELKHLLSRINHFEKI | 0.627 | 56.6 | 2.5 | 0.31 | WB | **-0.0548** |
| DRB1_0802 | RINHFEKIQIIPKNS | 0.6251 | 57.8 | 0.8 | 0.35 | SB | **0.8308** |
| DRB1_0901 | LSIYSTVASSLSLAI | 0.7162 | 21.6 | 1 | 0.54 | SB | **0.7452** |
| DRB1_1001 | TYQILSIYSTVASSL | 0.7431 | 16.1 | 2.5 | 0.65 | WB | **0.4829** |
| DRB1_1101 | EYAYKIVKKGDSAIM | 0.8593 | 4.6 | 0.12 | 0.81 | SB | **0.6036** |
| DRB1_1201 | INHFEKIQIIPKNSW | 0.6254 | 57.6 | 1.5 | 0.46 | SB | **0.5225** |
| DRB1_1301 | ELKHLLSRINHFEKI | 0.8771 | 3.8 | 0.4 | 0.45 | SB | **-0.0548** |
| DRB1_1302 | WTILKPNDAINFESN | 0.845 | 5.4 | 0.8 | 0.89 | SB | **0.4522** |
| DRB1_1501 | GNFIAPEYAYKIVKK | 0.6649 | 37.6 | 3.5 | 0.75 | WB | **0.2707** |
| DRB1_1602 | TYQILSIYSTVASSL | 0.738 | 17 | 0.4 | 0.71 | SB | **0.4829** |
| DRB3_0101 | KPLILRDCSVAGWLL | 0.6621 | 38.7 | 2.5 | 0.89 | WB | **-0.2793** |
| DRB3_0202 | CIGYHANNSTEQVDT | 0.7853 | 10.2 | 0.8 | 0.79 | SB | **0.5830** |
| DRB3_0301 | QTRLYQNPTTYISIG | 0.7837 | 10.4 | 3 | 0.75 | WB | **0.5228** |
| DRB4_0101 | LYDKVRLQLRDNAKE | 0.7005 | 25.6 | 0.9 | 0.65 | SB | **1.0144** |
| DRB4_0103 | KIVLLLAMVSLVKSD | 0.7796 | 10.9 | 2.5 | 0.43 | WB | **0.5485** |
| DRB5_0101 | ACPYLGSPSFFRNVV | 0.8705 | 4.1 | 0.25 | 0.96 | SB | **0.1034** |
| HLA-DQA10101-DQB10501 | LNKKMEDGFLDVWTY | 0.6171 | 63 | 1.3 | 0.67 | SB | **-0.0143** |
| HLA-DQA10102-DQB10501 | YQILSIYSTVASSLS | 0.8248 | 6.7 | 0.3 | 0.41 | SB | **0.5769** |
| HLA-DQA10102-DQB10502 | LLVLWGIHHPNDAAE | 0.5344 | 154.2 | 2.5 | 0.19 | WB | **0.2927** |
| HLA-DQA10102-DQB10602 | REEINGVKLESVGTY | 0.6445 | 46.8 | 0.7 | 0.88 | SB | **0.6718** |
| HLA-DQA10103-DQB10603 | QILSIYSTVASSLSL | 0.6627 | 38.5 | 0.01 | 0.38 | SB | **0.6037** |
| HLA-DQA10104-DQB10503 | QICIGYHANNSTEQV | 0.5141 | 192 | 0.6 | 0.64 | SB | **0.7618** |
| HLA-DQA10201-DQB10202 | YQILSIYSTVASSLS | 0.5924 | 82.3 | 0.08 | 0.67 | SB | **0.5769** |
| HLA-DQA10201-DQB10301 | SDHEASSGVSAACPY | 0.9168 | 2.5 | 0.08 | 0.44 | SB | **0.6057** |
| HLA-DQA10201-DQB10303 | ILSIYSTVASSLSLA | 0.7944 | 9.2 | 0.03 | 0.21 | SB | **0.6752** |
| HLA-DQA10201-DQB10402 | ILSIYSTVASSLSLA | 0.6609 | 39.2 | 0.8 | 0.38 | SB | **0.6752** |
| HLA-DQA10301-DQB10301 | KRGLFGAIAGFIEGG | 0.6366 | 51 | 1.2 | 0.31 | SB | **0.2706** |
| HLA-DQA10301-DQB10302 | VWTYNAELLVLMENE | 0.5698 | 105 | 0.25 | 0.47 | SB | **0.2402** |
| HLA-DQA10303-DQB10402 | FFRNVVWLIKKNSTY | 0.4001 | 659.4 | 5 | 0.64 | WB | **0.4780** |
| HLA-DQA10501-DQB10301 | PTTYISIGTSTLNQR | 0.6859 | 29.9 | 0.8 | 0.49 | SB | **1.0883** |
| HLA-DQA10501-DQB10302 | DHEASSGVSAACPYL | 0.5953 | 79.7 | 0.25 | 0.25 | SB | **0.6877** |
| HLA-DQA10501-DQB10303 | SDHEASSGVSAACPY | 0.6695 | 35.7 | 0.1 | 0.26 | SB | **0.6057** |
| HLA-DQA10501-DQB10402 | IMMAGLSLWMCSNGS | 0.6194 | 61.4 | 3.5 | 0.43 | WB | **-0.0891** |
| HLA-DQA10601-DQB10402 | YQILSIYSTVASSLS | 0.5689 | 106.1 | 1 | 0.32 | SB | **0.5769** |
| HLA-DPA10103-DPB10301 | YNSRFESVAWSASAC | 0.5605 | 116.2 | 2.5 | 0.64 | WB | **0.8230** |
| HLA-DPA10103-DPB10401 | GLDCIRPCFWVELIR | 0.5599 | 117 | 5.5 | 0.46 | WB | **0.9088** |
| HLA-DPA10103-DPB10402 | DCIRPCFWVELIRGR | 0.3542 | 1083.3 | 0.7 | 0.67 | SB | **0.6870** |
| HLA-DPA10103-DPB10601 | IRPCFWVELIRGRPK | 0.7863 | 10.1 | 3.5 | 0.49 | WB | **0.9475** |
| HLA-DPA10201-DPB10101 | LDCIRPCFWVELIRG | 0.5231 | 174.2 | 6.5 | 0.36 | WB | **0.6113** |
| HLA-DPA10201-DPB10501 | GQASYKIFKMRKGKV | 0.4556 | 361.6 | 7.5 | 0.29 | WB | **0.1931** |
| HLA-DPA10201-DPB11401 | CRIFFLTQGALMNDK | 0.4745 | 294.6 | 2.5 | 0.43 | WB | **0.4735** |
| HLA-DPA10301-DPB10402 | IRPCFWVELIRGRPK | 0.6392 | 49.6 | 2.5 | 0.65 | WB | **0.9475** |
| HLA-DPA10103-DPB10201 | HLECRIFFLTQGALM | 0.6416 | 48.3 | 3 | 0.24 | WB | **0.9001** |

**Table S6.** HTL cell epitopes predicted from NA protein

| Allele | peptide | 1-log50k(aff) | affinity(nM) | %Rank | Relia | Bind | Vaxijen |
| --- | --- | --- | --- | --- | --- | --- | --- |
| DRB1_0101 | RIFFLTQGALMNDKH | 0.8928 | 3.2 | 0.12 | 0.67 | SB | **0.6793** |
| DRB1_0103 | ASYKIFKMRKGKVVK | 0.2999 | 1949.2 | 0.6 | 0.35 | SB | **-0.3331** |
| DRB1_0301 | VVKSVELDAPNYHYE | 0.5197 | 180.7 | 7.5 | 0.88 | WB | **0.5807** |
| DRB1_0401 | GSCFTVMTDGPSNGQ | 0.5882 | 86.1 | 4.5 | 0.75 | WB | **-0.3983** |
| DRB1_0402 | VKGFSFKYGNGVWIG | 0.4704 | 308.1 | 0.4 | 0.25 | SB | **0.7510** |
| DRB1_0403 | TIGSICMVIGMVSLM | 0.4479 | 392.8 | 0.1 | 0.38 | SB | **1.4483** |
| DRB1_0404 | GNIISIWVSHPIQKG | 0.7473 | 15.4 | 0.4 | 0.46 | SB | **0.0235** |
| DRB1_0405 | CRIFFLTQGALMNDK | 0.6322 | 53.5 | 3 | 0.46 | WB | **0.4735** |
| DRB1_0701 | IFKMRKGKVVKSVEL | 0.7897 | 9.7 | 0.9 | 0.67 | SB | **0.2956** |
| DRB1_0801 | ASYKIFKMRKGKVVK | 0.708 | 23.6 | 0.3 | 0.37 | SB | **-0.3331** |
| DRB1_0802 | GNIISIWVSHPIQKG | 0.5678 | 107.4 | 2.5 | 0.33 | WB | **0.0235** |
| DRB1_0901 | VKGFSFKYGNGVWIG | 0.7495 | 15 | 0.5 | 0.84 | SB | **0.7510** |
| DRB1_1001 | RIFFLTQGALMNDKH | 0.73 | 18.6 | 3 | 0.41 | WB | **0.6793** |
| DRB1_1101 | ASYKIFKMRKGKVVK | 0.8924 | 3.2 | 0.04 | 0.36 | SB | **-0.3331** |
| DRB1_1201 | QASYKIFKMRKGKVV | 0.6002 | 75.6 | 2.5 | 0.75 | WB | **0.0581** |
| DRB1_1301 | YKIFKMRKGKVVKSV | 0.8543 | 4.8 | 0.7 | 0.25 | SB | **-0.4643** |
| DRB1_1302 | GMVSLMLQIGNIISI | 0.7714 | 11.9 | 2.5 | 0.65 | WB | **0.9942** |
| DRB1_1501 | IGNIISIWVSHPIQK | 0.8321 | 6.2 | 0.17 | 0.64 | SB | **-0.2777** |
| DRB1_1602 | SYKIFKMRKGKVVKS | 0.7117 | 22.6 | 0.7 | 0.38 | SB | **-0.3653** |
| DRB3_0101 | ECRIFFLTQGALMND | 0.5406 | 144.1 | 6.5 | 0.64 | WB | **0.3307** |
| DRB3_0202 | IKSWRNNILRTQESE | 0.6291 | 55.3 | 4 | 0.59 | WB | **-0.5418** |
| DRB3_0301 | VSLMLQIGNIISIWV | 0.8753 | 3.9 | 0.3 | 0.6 | SB | **0.4721** |
| DRB4_0101 | KGKVVKSVELDAPNY | 0.6542 | 42.2 | 2 | 0.7 | WB | **0.2235** |
| DRB4_0103 | SYKIFKMRKGKVVKS | 0.8803 | 3.7 | 0.2 | 0.28 | SB | **-0.3653** |
| DRB5_0101 | ASYKIFKMRKGKVVK | 0.8111 | 7.7 | 1 | 0.31 | SB | **-0.3331** |
| HLA-DQA10101-DQB10501 | LCPIRGWAVHSKDNN | 0.5095 | 201.8 | 5 | 0.74 | WB | **0.6944** |
| HLA-DQA10102-DQB10501 | ICMVIGMVSLMLQIG | 0.8307 | 6.2 | 0.2 | 0.46 | SB | **1.5671** |
| HLA-DQA10102-DQB10502 | LCPIRGWAVHSKDNN | 0.413 | 573 | 11 | 0.35 |  | **0.6944** |
| HLA-DQA10102-DQB10602 | IKSWRNNILRTQESE | 0.6003 | 75.6 | 1.6 | 0.49 | SB | **-0.5418** |
| HLA-DQA10103-DQB10603 | GSICMVIGMVSLMLQ | 0.5508 | 129.1 | 0.5 | 0.54 | SB | **1.2727** |
| HLA-DQA10104-DQB10503 | LCPIRGWAVHSKDNN | 0.4262 | 497.2 | 3 | 0.77 | WB | **0.6944** |
| HLA-DQA10201-DQB10202 | ECRIFFLTQGALMND | 0.3493 | 1142.3 | 9.5 | 0.41 | WB | **0.3307** |
| HLA-DQA10201-DQB10301 | KESTIWTSGSSISFC | 0.8618 | 4.5 | 0.5 | 0.34 | SB | **0.0804** |
| HLA-DQA10201-DQB10303 | NSRFESVAWSASACH | 0.7363 | 17.3 | 0.25 | 0.5 | SB | **0.7947** |
| HLA-DQA10201-DQB10402 | QASYKIFKMRKGKVV | 0.7965 | 9 | 0.05 | 0.57 | SB | **0.0581** |
| HLA-DQA10301-DQB10301 | STIWTSGSSISFCGV | 0.669 | 35.9 | 0.6 | 0.37 | SB | **-0.1835** |
| HLA-DQA10301-DQB10302 | RIGSKGDVFVIREPF | 0.5184 | 183.2 | 0.8 | 0.51 | SB | **0.4192** |
| HLA-DQA10303-DQB10402 | ASYKIFKMRKGKVVK | 0.6864 | 29.8 | 0.06 | 0.56 | SB | **-0.3331** |
| HLA-DQA10401-DQB10402 | SRFESVAWSASACHD | 0.3915 | 723.3 | 8 | 0.41 | WB | **0.5470** |
| HLA-DQA10501-DQB10201 | AVAVLKYNGIITDTI | 0.534 | 154.8 | 3.5 | 0.69 | WB | **0.1530** |
| HLA-DQA10501-DQB10301 | STIWTSGSSISFCGV | 0.6447 | 46.7 | 1.7 | 0.29 | SB | **-0.1835** |
| HLA-DQA10501-DQB10302 | SRFESVAWSASACHD | 0.6071 | 70.2 | 0.15 | 0.26 | SB | **0.5470** |
| HLA-DQA10501-DQB10303 | SRFESVAWSASACHD | 0.6451 | 46.5 | 0.3 | 0.24 | SB | **0.5470** |
| HLA-DQA10501-DQB10402 | SLCPIRGWAVHSKDN | 0.8173 | 7.2 | 0.05 | 0.46 | SB | **0.7153** |
| HLA-DQA10601-DQB10402 | QASYKIFKMRKGKVV | 0.7467 | 15.5 | 0.03 | 0.53 | SB | **0.0581** |
| DRB1_1301 | YKIFKMRKGKVVKSV | 0.8543 | 4.8 | 0.7 | 0.25 | SB | **-0.4643** |
| DRB1_1302 | GMVSLMLQIGNIISI | 0.7714 | 11.9 | 2.5 | 0.65 | WB | **0.9942** |
| DRB1_1501 | IGNIISIWVSHPIQK | 0.8321 | 6.2 | 0.17 | 0.64 | SB | **-0.2777** |
| DRB1_1602 | SYKIFKMRKGKVVKS | 0.7117 | 22.6 | 0.7 | 0.38 | SB | **-0.3653** |
| DRB3_0101 | ECRIFFLTQGALMND | 0.5406 | 144.1 | 6.5 | 0.64 | WB | **0.3307** |
| DRB3_0202 | IKSWRNNILRTQESE | 0.6291 | 55.3 | 4 | 0.59 | WB | **-0.5418** |
| DRB3_0301 | VSLMLQIGNIISIWV | 0.8753 | 3.9 | 0.3 | 0.6 | SB | **0.4721** |
| DRB4_0101 | KGKVVKSVELDAPNY | 0.6542 | 42.2 | 2 | 0.7 | WB | **0.2235** |
| DRB4_0103 | SYKIFKMRKGKVVKS | 0.8803 | 3.7 | 0.2 | 0.28 | SB | **-0.3653** |
| DRB5_0101 | ASYKIFKMRKGKVVK | 0.8111 | 7.7 | 1 | 0.31 | SB | **-0.3331** |
| HLA-DPA10103-DPB10301 | YNSRFESVAWSASAC | 0.5605 | 116.2 | 2.5 | 0.64 | WB | **0.8230** |
| HLA-DPA10103-DPB10401 | GLDCIRPCFWVELIR | 0.5599 | 117 | 5.5 | 0.46 | WB | **0.9088** |
| HLA-DPA10103-DPB10402 | DCIRPCFWVELIRGR | 0.3542 | 1083.3 | 0.7 | 0.67 | SB | **0.6870** |
| HLA-DPA10103-DPB10601 | IRPCFWVELIRGRPK | 0.7863 | 10.1 | 3.5 | 0.49 | WB | **0.9475** |
| HLA-DPA10201-DPB10101 | LDCIRPCFWVELIRG | 0.5231 | 174.2 | 6.5 | 0.36 | WB | **0.6113** |
| HLA-DPA10201-DPB10501 | GQASYKIFKMRKGKV | 0.4556 | 361.6 | 7.5 | 0.29 | WB | **0.1931** |
| HLA-DPA10201-DPB11401 | CRIFFLTQGALMNDK | 0.4745 | 294.6 | 2.5 | 0.43 | WB | **0.4735** |
| HLA-DPA10301-DPB10402 | IRPCFWVELIRGRPK | 0.6392 | 49.6 | 2.5 | 0.65 | WB | **0.9475** |
| HLA-DPA10103-DPB10201 | HLECRIFFLTQGALM | 0.6416 | 48.3 | 3 | 0.24 | WB | **0.9001** |

**Table S7.** Nucleotide sequence of 'H5N1 construct' optimized codon

| ATGAGGACACTAGACTTTCACGATTCAAATAAGAAGAACAGCTCGTTATGCCCGATTAAAAAGAAGTCCGATCAGATCTGCATTGGTTATGCTGCGTACTGCCCGTATTTGGGTAGTCCGAGCTTCGCGGCGTACCGCCTGGTACCGAAAATCGCAACCCGTGCAGCCTATCAAAGCGGTCGTATGGAATTTTTCTGGCTGGCTGCTTACATGCCGTTTCACAACATCCATCCGCTGACCATCGCCGCGTACATCACCATTGGCTCTATCTGCATGGTGGCCGCGTACATGGTTATTGGCATGGTCAGCCTGATGGCAGCTTACCTGCAAATCGGCAATATCATCAGCATTGCGGCTTACCATCTGGAGTGCCGCATTTTTTTCCTTGCCGCTTACTTGGAGTATCAGATCGGCTATATTTGCTCCGGTGCGGCCTACGCATACGGCGTTAAGGGTTTTAGCTTTAAATACGCGGCGTACGGCCTGGACTGTATTCGTCCGTGTTTTGGTCCAGGACCGGGCCCGGGCCCTACGACCTATATTTCTATCGGCACCAGCACGCTGAACCAGCGTGGTCCAGGTCCGGGTCCGGGGGCAGAGCTGTTAGTGCTGATGGAAAATGAACGTACCTTGGACTTCCACGGCCCGGGCCCAGGTCCGGGCAACCTGTATGACAAAGTGCGCCTGCAGCTGCGTGATAATGCAAAGGAGCACGAATATGGCGCAGAAGCCTTGGAGCGCGCGGGTATGCGTATCCACTATCTTCTGTTCGCCCTGTTGTTCCTCTTCTTGGTGCCGGTTCCGGGTCATGGTGGCATTATCAACACCTTGCAAAAATATTACTGCCGTGTTCGTGGTGGTCGCTGCGCAGTTCTGAGCTGTCTGCCGAAAGAGGAACAGATTGGTAAATGCTCCACTCGCGGTCGTAAATGTTGTAGACGTAAAAAGGAGGCGGCGGCTAAGGCAAAGTTCGTGGCGGCGTGGACCCTGAAAGCAGCGGCCGAAGCTGCGGCGAAGGAGGCGGCGGCGAAG |
| --- |
